# N_2_O Activation and NO Adsorption Control the Simultaneous Conversion of N_2_O and NO Using NH_3_ over Fe-ZSM-5

**DOI:** 10.1021/jacs.5c01100

**Published:** 2025-02-28

**Authors:** Filippo Buttignol, Alberto Garbujo, Pierdomenico Biasi, Oliver Kröcher, Davide Ferri

**Affiliations:** † Paul Scherrer Institute, PSI Center for Energy and Environmental Sciences, CH-5232 Villigen, Switzerland; ‡ Institute for Chemical Sciences and Engineering, École polytechnique fédérale de Lausanne (EPFL), CH-1015 Lausanne, Switzerland; § Basic Research Department, Casale SA, CH-6900 Lugano, Switzerland

## Abstract

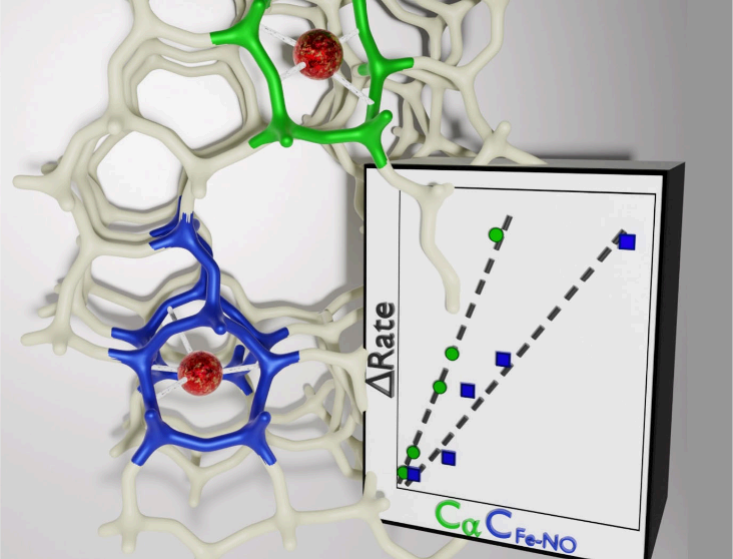

Fe-exchanged zeolites are heterogeneous catalysts that can potentially ensure simultaneous conversion of nitrous oxide (N_2_O) and nitric oxide (NO) using ammonia (NH_3_) as a selective reducing agent through their selective catalytic reduction reaction (N_2_O-NO-SCR). In this study, we rationalize the origin of the beneficial effect of N_2_O on the NO conversion by combining catalytic experiments with *ex situ* characterization and in *situ/operando* X-ray absorption spectroscopy (XAS) and infrared spectroscopy in diffuse reflectance mode (DRIFTS) on a series of Fe-ZSM-5 catalysts where we attempted to control Fe speciation at constant Fe content. The catalytic activity data revealed that N_2_O can promote NO conversion at different temperatures and to different extents. This behavior was found to be related to the activity of the catalysts in the NO-mediated N_2_O decomposition reaction, which ensures the oxidative transformation of NO and thus sustains the N_2_O-NO-SCR chemistry. The oxidation activity is in turn determined by processes of N_2_O activation and NO adsorption, which are a function of the Fe speciation and are likely catalyzed by a minority of isolated Fe^2+^ sites coordinated in different cationic environments. In agreement, the concentrations of the Fe species able to activate N_2_O (*C*_α_) and of the Fe species able to coordinate NO (*C*_Fe-NO_) decrease with an increasing degree of Fe agglomeration and govern especially the promotion of the NO conversion induced by N_2_O in this dual-site mechanism. Maximization of the concentration of both species is therefore essential to design Fe-exchanged zeolites with the highest activity toward the N_2_O-NO-SCR reaction.

## Introduction

Exhaust after-treatment catalysis is a very mature field of research, inevitably losing the past broad scientific interest due to the decline of internal combustion engines for automotive applications in favor of electric vehicles. However, satellite images taken during the coronavirus lockdown in 2020 and shortly after it in the same year clearly demonstrate that emissions of pollutants from industrial and mobile sources are still a concern in many regions of the globe including developed countries.^1^ Especially nitrous oxide (N_2_O) and nitric oxides (NO and NO_2_) contribute significantly to environmental issues such as photochemical smog, acid rains, and climate change.^1,2^ N_2_O emissions, in particular, need to be controlled due to its tremendous global warming potential exceeding by far that of more openly discussed carbon dioxide. In the past decades, a large research effort has mainly focused on the individual conversion of NO_*x*_ and N_2_O exploiting different redox mediated reactions catalyzed by very similar heterogeneous catalysts based on Fe-exchanged zeolites.^3,4^

The mitigation of N_2_O emissions^4^ occurs via a two-step process comprising an oxidation and a reduction half-cycle (OHC and RHC, respectively). First, N_2_O undergoes the so-called activation (eq 1), i.e., the OHC.^5^ Upon interaction through the O atom with the reduced (2+) form of an active Fe center (α), the N_2_–O bond is cleaved, N_2_ is released, and an oxygen atom is left behind promoting Fe site oxidation and formation of α-O species.^6^
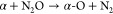
1In order to close the catalytic cycle, α-O needs to be reduced. Under N_2_O decomposition conditions, the RHC is rate-limiting and consists of the formation of O_2_ via a surface migration and recombination process of α-O at sufficiently high temperature (eq 2):^7^
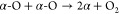
2

Thus, it is not surprising that reducing agents such as NO or NH_3_ promote N_2_O conversion by interacting with the α-O species according to the NO-mediated N_2_O decomposition^8^ and N_2_O-SCR^9^ reactions.

The individual conversion of NO is instead achieved in the presence of NH_3_ according to the selective catalytic reduction (NO-SCR) scheme (eq 3) in which an O_2_-rich atmosphere ensures reoxidation of the active Fe sites to promote the rate-determining step, i.e., the oxidative activation of NO.^10,11^
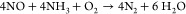
3

Contrarily to these processes, the simultaneous conversion of N_2_O and NO using NH_3_ under SCR conditions (N_2_O-NO-SCR) has been largely disregarded and only a handful of research exists.^9,12−15^ Nevertheless, the N_2_O-NO-SCR technology is appealing for all the applications in which both N_2_O and NO are present together in the flue gases^16,17^ and is recognized as a potential solution for exhaust treatment of rising combustion technologies such as NH_3_ internal combustion engines for the maritime field.^16,18^ Early experiments highlighted that the NO conversion was promoted in the presence of N_2_O and the extent of promotion was catalyst dependent.^9,13^ Successive studies reported milder promotion of N_2_O on the NO conversion.^14^ On the contrary, a moderate or even detrimental effect of N_2_O was recently reported depending on the Fe-exchanged zeolite employed as well as on the temperature regime.^15^

Given this limited and controversial knowledge, we recently performed a fundamental study to rationalize the reaction mechanism of the N_2_O-NO-SCR reaction over a commercial Fe-FER catalyst.^19^ The technical relevance of the formulation of this sample, including a binder and possibly additives, did not allow us to have control over the properties of the material. However, we demonstrated that N_2_O-NO-SCR conditions yield superior conversions of both N_2_O and NO compared to the individual reactions. This enhanced performance arises from the cooperation between the redox processes involving distinct Fe^2+^/Fe^3+^ pairs responsible for the individual transformations of N_2_O and NO. N_2_O aids the NO-SCR reaction by supplying reactive oxidizing hydroxyl groups and favoring NO oxidation, while NO-SCR provides an efficient reduction half-cycle driving enhanced N_2_O decomposition. We also concluded that N_2_O favors NO conversion according to a dual-site mechanism in which isolated square-planar Fe^2+^ sites in β-cationic position ensure N_2_O activation, while isolated tetrahedrally coordinated Fe^2+^ species in γ-cationic position are required for adsorption and successive redox mediated oxidation of NO prior to interaction with NH_3_.

Besides Fe-FER also catalysts based on Fe-ZSM-5 are deployed for the remediation of N_2_O and NO.^20^ Hence, in order to broaden the understanding of their concomitant conversion, to attempt at generalizing the rationale obtained on commercial Fe-FER to a different zeolite topology, and to determine if material properties such as Fe speciation control the effect of N_2_O on the NO conversion, the N_2_O-NO-SCR reaction was studied over a series of Fe-ZSM-5 catalysts.

It is recognized that the introduction of Fe ions in zeolite materials is a complex procedure, generally leading to a wide distribution of Fe species.^21,22^ Fe atoms in extra-framework positions can indeed exist in the form of (i) isolated Fe^2+^ or Fe^3+^ sites possessing different coordination to framework oxygen atoms and possibly located in different cationic positions, (ii) oligomeric Fe_*x*_O_*y*_(OH)_*z*_ species possessing various degrees of agglomeration, and (iii) bulk Fe_*x*_O_*y*_ particles of varying sizes and crystallinity on the outermost part of the porous structure.^23−26^ A good dispersion in the form of monomeric species can often be achieved at low Fe content.^23^ Vice versa, more agglomerated species are generally present in catalysts with high Fe loading.^22^ Nevertheless, it is arduous to obtain catalysts with the same Fe loading but different Fe speciation for a systematic study since, at constant Fe content, there is only limited possibility for controlling the fraction of the various species during the synthesis.

In this work, we prepared a series of Fe-ZSM-5 catalysts with identical Fe content and increasing degree of agglomeration by tuning the calcination conditions of a (noncalcined) parent material possessing homogeneous Fe dispersion in the form of isolated Fe sites. Combining catalytic experiments with *ex situ* characterization and *in situ/operando* spectroscopies, we derived quantitative structure–activity relationships that we were not able to obtain on the previous commercial Fe-FER catalyst. These results prove that the concentrations of the Fe species able to activate N_2_O and of the Fe species able to coordinate NO govern the extent of promotion of the NO conversion by N_2_O. The results indicate that N_2_O activation and NO oxidative transformation as well as the related concentration of isolated Fe sites in β- and γ-cationic positions are fundamental parameters for maximizing the catalyst activity in the simultaneous conversion of N_2_O and NO using NH_3_.

## Experimental Section

### Materials

The noncalcined Fe-ZSM-5 (2.4 wt % Fe, Si/Al = 12.2) with homogeneous Fe dispersion in the form of isolated Fe sites (Figure S1) was provided by Casale S.A. and was previously characterized.^27^ Five derivatives of this material were obtained by adopting progressively harsher calcination conditions. Samples labeled FeZ-500R and FeZ-800R were obtained by calcination (8 h) in a muffle oven with a temperature ramp of 2 °C/min from room temperature to 500 and 800 °C, respectively. The lowest calcination temperature (500 °C) was selected based on the need to test catalysts at elevated temperatures for effective occurrence of N_2_O-NO-SCR. When the noncalcined Fe-ZSM-5 was introduced into the hot muffle oven at 800, 850, or 900 °C (8 h), samples were labeled FeZ-800, FeZ-850, and FeZ-900, respectively. Therefore, we consider that the severity of the calcination treatment increases in the order: FeZ-500R < FeZ-800R < FeZ-800 < FeZ-850 < FeZ-900.

### Characterization

Powder X-ray diffraction (XRD) patterns were collected on a D8ADVANCE (Bruker) diffractometer using Cu Kα1 radiation (λ = 1.5406 Å). Data were recorded from 5° to 65° 2θ at a step size of 0.02°/s.

The apparent specific surface area of the catalysts (*S*_a_) was measured by Ar physisorption at −186 °C with a Quantachrome Autosorb iQ instrument equipped with the CryoSync accessory allowing the temperature of liquid N_2_ to be maintained at the liquefaction point of Ar. Prior to analysis, the catalysts were evacuated under reduced pressure at 350 °C for 12 h in order to remove adsorbed water. The apparent specific surface area was determined using the Brunauer–Emmett–Teller (BET) method. The pore-size distribution was derived from the adsorption branch of the isotherm according to the nonlocal density functional theory (NLDFT) for Ar adsorption at −186 °C in cylindrical pores of zeolite materials.

Ultraviolet–visible spectra were recorded in diffuse reflectance mode (DRUV) by using a spectrometer (Varian Cary 4000) equipped with a diffuse reflectance accessory (Praying Mantis, Harrick) at 25 °C after dehydration of the sample in a flow of 100 mL·min^–1^ N_2_ at 400 °C for 30 min. The parent ZSM-5 material was used as reference. All the spectra were converted into the Kubelka–Munk function. Deconvolution of the spectra in Gaussian bands was performed using the OriginLab 2021b software. Estimation of fractions of iron species was obtained by dividing the area of peak *i* obtained from deconvolution and the total area, *A*_tot_, to obtain the area ratio *A*_*i*_/*A*_tot_.

X-band electron paramagnetic resonance (EPR) measurements were performed at −243 and 25 °C using a spectrometer (Bruker ELEXYS E500) equipped with a microwave bridge (SuperX EPR049) and a cylindrical cavity (TE011 ER 4122SHQE) in connection with a continuous flow cryostat (Oxford Instruments). The samples were dehydrated under a vacuum at 150 °C for 6 h prior to analysis. The following parameters were used for all measurements: microwave frequency, 9.371147 GHz; microwave power, 6.4 mW; modulation frequency, 100 kHz; modulation amplitude, 5 G; detector receiving gain, 54 dB; time constant, 10.24 ms; conversion time, 40.96 ms; and spectral resolution, 1024 data points.

X-ray absorption spectroscopy (XAS) measurements were performed in transmission mode at the Fe K edge (7.112 keV) at the SuperXAS beamline of the Swiss Light Source (SLS, Switzerland). The samples (40 mg) were mixed with cellulose (10 mg) and pressed into pellets. X-ray absorption near edge structure (XANES) normalization and extended X-ray absorption fine structure (EXAFS) extraction (3 ≤ *k*-range ≤ 10.5) were conducted using the Athena software.^28^

Temperature-programmed desorption (NH_3_-TPD) experiments were conducted in a lab-scale experimental setup comprising mass flow controllers for precise dosing of gases and an FTIR spectrometer (ThermoFisher Antaris) equipped with a heated 2-m gas cell for gas concentration analysis. All experiments were performed at atmospheric pressure, and the reaction temperature was monitored and controlled by a K-type thermocouple inserted in the catalyst bed. In each test, the sample (50 mg) was mixed with 50 mg of cordierite and loaded into a tubular quartz reactor (*d*_i_ = 6 mm). After dehydration in a flow of N_2_ at 500 °C for 30 min, the catalysts were exposed to 500 ppm of NH_3_/N_2_ at 150 °C for 90 min, followed by N_2_ flush at 150 °C for 90 min to induce desorption of physisorbed NH_3_. Finally, chemisorbed NH_3_ was desorbed by using a temperature ramp (10 °C·min^–1^) from 150 to 600 °C under N_2_. The total gas flow was kept at 100 mL·min^–1^ throughout the whole experiment.

### Catalytic Measurements

The powder catalysts were coated on cordierite honeycomb of size 1.2 cm × 1.7 cm × 5.1 cm with a cell density of 400 cpsi to perform catalytic measurements. The washcoating was carried out by immersing the cordierite core in a suspension of ca. 15 g of the powder catalyst (particle dimension smaller than 10 μm) and 10 wt % of binder (LUDOX AS-40 SiO_2_) with respect to the catalyst weight in distilled water. This procedure was repeated until the desired catalyst loading (ca. 0.4 g) was achieved followed by calcination in air at 500 °C for 4 h (2 °C·min^–1^).

Catalytic activity measurements were performed at atmospheric pressure between 200 and 500 °C at a total flow rate of 800 NL·h^–1^ at STP in a setup described elsewhere.^29^ The setup comprised a monolithic Pt/Al_2_O_3_ catalyst for in situ water generation through precise flows of H_2_ and O_2_. All gas lines were heated to 150 °C in order to avoid condensation. The washcoated catalysts were tested for NO_*x*_ and N_2_O conversions (χ) under a broad range of conditions (Table 1) and were calculated according to eq 4:
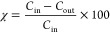
4where *C*_in_ and *C*_out_ are the concentrations (ppm) of NO_*x*_ or N_2_O in the inlet and outlet gas, respectively. Tests were carried out with an excess dosage of NH_3_ ([NH_3_]/[NO_*x*_] = 1.2) in order to prevent limitation because of insufficient concentration of reducing agent. An insufficient supply of NH_3_ greatly limits the improvement of the NO conversion by N_2_O, which is mirrored by the evolution of NO_2_.^19^

**Table 1:** Feed Compositions of Catalytic Experiments

Experiment	NO (ppm)	N_2_O (ppm)	NH_3_ (ppm)	O_2_ (vol %)	H_2_O (vol %)
N_2_O-NO-SCR	500	500	600	3	0.3
NO-SCR	500	0	600	3	0.3
NO-mediated N_2_O decomposition	500	500	0	3	0.3
N_2_O decomposition	0	500	0	3	0.3

The normalized reaction rates (*r*, μmol·g_cat_^–1^·s^–1^) for NO_*x*_ and N_2_O conversion were calculated using eq 5:
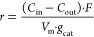
5where *C*_in_ and *C*_out_ are the concentrations (ppm) of NO_*x*_ or N_2_O in the inlet and outlet gas, respectively, *F* is the total flow rate (L·s^–1^), *V*_m_ is the molar volume (mL·mmol^–1^), and *g*_cat_ is the catalyst mass (g) washcoated on the honeycomb. The increase in *r* for NO_*x*_ conversion in the presence of N_2_O (Δ*r*_N_2_O-NO-SCR_) was derived from eq 6:
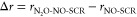
6where *r*_N_2_O-NO-SCR_ is *r* for NO_*x*_ conversion under N_2_O-NO-SCR conditions and *r*_NO-SCR_ is *r* for NO_*x*_ conversion under NO-SCR.

### In Situ and Operando Characterization

*In situ* and *operando* diffuse reflectance infrared Fourier transform spectroscopy (DRIFTS) and *operando* X-ray absorption spectroscopy (XAS) experiments were conducted using the same experimental setup comprising mass flow controllers (Bronkhorst) and solenoid valves (Series 9 and Parker) to control the gas flows and automatically switch between gases, respectively. In all experiments, ca. 40 mg of sieved catalyst (100–150 μm) was loaded in custom-built spectroscopic cells for DRIFTS^30^ and XAS^31^ experiments. A mass spectrometer (Omnistar, Pfeiffer) connected downstream of the cell was used for online monitoring of the outlet gas concentrations, *m*/*z* = 17 (NH_3_), 18 (H_2_O), 28 (N_2_), 30 (NO), 32 (O_2_), 40 (Ar), and 44 (N_2_O). In order to account for possible fluctuations in the total flow rate, after each experiment, the ion currents of the mass fragment of interest were normalized by the ion current of Ar, i.e., the inert gas.

DRIFT spectra were acquired at a resolution of 4 cm^–1^ by accumulating 100 (background) and 10 (sample) interferograms at a scanner velocity of 80 kHz by using a Vertex 70 spectrometer (Bruker) equipped with a diffuse reflection accessory (Praying Mantis, Harrick).

*Operando* XAS experiments were performed at the SuperXAS beamline of the Swiss Light Source facility (SLS, Switzerland). A Si-coated mirror was used to collimate the X-ray polychromatic beam resulting from a 2.9 T bending magnet. Monochromatic light was obtained through a channel cut Si(311) liquid N_2_-cooled monochromator, which also allowed collection in quick-scanning extended X-ray absorption fine-structure spectroscopy (QEXAFS) mode at 1 Hz.^32^ The X-ray beam (1 × 0.2 mm) was successively filtered using a 40 μm Al filter in order to avoid possible undesired phenomena such as hot-spots^10^ and X-ray induced reduction of Fe centers.^33^ Measurements were performed in fluorescence mode at the Fe K edge (7.112 keV) using a passivated implanted planar Si (PIPS) detector.^34^ Two quartz wool plugs were used to fix the catalyst bed between two graphite windows (0.5 mm thick). A K-type thermocouple inserted into the spectroscopic cell (ca. 1 mm aside from the catalyst bed) was used to monitor and control the reaction temperature. For absolute energy calibration, the stainless steel body of the custom-made cell was measured for 20 s before the beam was focused onto the catalyst bed. The raw XAS spectra were processed, calibrated, and normalized using a python-based processing software for QEXAFS data.^35^ Prior to each experiment, the catalyst was dehydrated in situ in a flow of 5 vol % O_2_ balanced in Ar at 500 °C (10 °C·min^–1^) for 30 min. A total flow rate of 100 mL·min^–1^ was used for pretreatment and successive tests.

*In situ* NH_3_ adsorption–desorption experiments were conducted by saturating the dehydrated catalysts with 1000 ppm of NH_3_/Ar at 150 °C for 15 min followed by flushing in Ar for ca. 20 min to allow the desorption of physisorbed NH_3_. DRIFT spectra were collected at the end of this desorption step to mimic NH_3_-TPD experiments.

*In situ* N_2_O addition experiments were performed by introducing 1000 ppm of N_2_O/Ar for 5 min over the dehydrated FeZ-500R catalyst while recording DRIFT spectra.

*Operando* experiments were conducted according to the modulated excitation (ME) approach^36^ at 400 °C. In the case of DRIFTS, they consisted of the repeated addition and cutoff of (i) 1000 ppm of N_2_O/Ar in a constant flow of 1000 ppm of NO/Ar and (ii) 1000 ppm of NO/Ar in a constant flow of Ar. Pulses were performed every 240 s for 2 h (30 pulses) while recording a total of 376 spectra in one full period (1 spectrum = ca. 1.27 s). In the case of XAS, the pulse sequence consisted of addition and cutoff of 1000 ppm of N_2_O/Ar over the FeZ-500R, FeZ-800R, or FeZ-800 catalysts in a constant flow of 1000 ppm of NO, 1000 ppm of NH_3_, and 3 vol % O_2_/Ar. Pulses were performed every 120 s for 2 h (60 pulses) while recording a total of 480 spectra in one full period (1 spectrum of 0.5 s).

In order to isolate the contribution of the actual active species from the larger fraction of stationary species, the time-resolved DRIFT and XAS spectra obtained in the ME experiments were averaged and subjected to phase sensitive detection (PSD) to obtain the corresponding phase-resolved spectra as described elsewhere.^36,37^ A custom-made Matlab script was used to process the spectra by averaging and PSD. The phase-domain DRIFT and XAS spectra display only the signals of species that reversibly responded to the perturbation, i.e., the N_2_O pulses. The signals related to spectator or nonresponsive species are suppressed, thus enhancing signals associated with active species. Signals of opposite sign in the phase-resolved spectra indicate that the signals do not belong to the same species and that the related species are likely interconverted. Differently, if the signals possess the same sign various explanations are possible. If the variation of their intensity is coherent, the signals can be related to the same species (possessing multiple spectral fingerprints) or different species possessing the same kinetic behavior. If their behavior is not coherent, the signals relate to two species exhibiting different kinetics but that are still produced or consumed in the same half-cycle.^38^

The concentrations of the Fe sites capable of activating N_2_O (α-sites, *C*_α_) and of the Fe sites able to adsorb NO (*C*_Fe-NO_) were determined by employing the same experimental setup used for DRIFTS and XAS measurements and with ca. 40 mg of catalyst in the same cell used in the DRIFTS experiments.^30^*C*_α_ was measured following a similar procedure to that established by Panov and co-workers.^39^ In each experiment, the sample was treated in situ in a flow of 5 vol % O_2_/Ar (100 mL·min^–1^) at 500 °C for 30 min (10 °C·min^–1^). Then, the sample was cooled in Ar to 200 °C and was exposed to a flow of 1000 ppm of N_2_O/Ar (100 mL·min^–1^) for 15 min. At this temperature, the N_2_O decomposition reaction is limited by the RHC; i.e., no O_2_ evolves from the catalyst, and saturation of the α-sites by N_2_O occurs according to eq 1. *C*_α_ (sites·g_cat_^–1^) is then calculated according to eq 7:
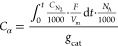
7where *C*_N_2__ is the N_2_ concentration (ppm), *F* is the total flow rate (L·min^–1^), *V*_m_ is the molar volume (mL·mmol^–1^), *N*_a_ is Avogadro’s number (mol^–1^), and *g*_cat_ the catalyst mass (g). In practice, we integrated the transient N_2_ production peak monitored by the online MS (*m*/*z* = 44) by taking into account the 1:1 stoichiometry between N_2_ production and α-sites and the amount of catalyst used in the experiment. For an accurate *C*_α_ calculation, the N_2_ signal was calibrated after each experiment.

For quantification of *C*_Fe-NO_, the catalysts were treated in a flow of Ar at 500 °C for 30 min (10 °C·min^–1^). After treatment, the catalyst was cooled to 200 °C and exposed to 250 ppm of NO/Ar for ca. 15 min to achieve saturation of the Fe sites with NO. A total flow rate of 100 mL·min^–1^ was used for pretreatment and successive tests. *C*_Fe-NO_ was calculated according to eq 8:
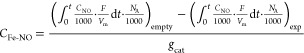
8where *C*_NO_ is the NO concentration (ppm), *F* is the total flow rate (L·min^–1^), *V*_m_ is the molar volume (mL·mmol^–1^), *N*_a_ is Avogadro’s number (mol^–1^), and *g*_cat_ is the catalyst mass (g). In practice, *C*_Fe-NO_ is calculated by integration of the NO signal monitored by the online MS (*m*/*z* = 30) until catalyst saturation, by the difference between the value derived from a reference experiment conducted with the empty spectroscopic cell (empty) and the value during the actual experiment (exp), by taking into account the 1:1 adsorption stoichiometry between NO and Fe and the amount of catalyst used in the experiment. For an accurate *C*_Fe-NO_ calculation, the NO signal was calibrated after each experiment. Saturation of the catalysts with NO was simultaneously monitored by DRIFT spectroscopy by recording spectra, as reported above for the *in situ* experiments.

## Results and Discussion

### Catalyst Characterization

All the Fe-ZSM-5 catalysts displayed the characteristic peaks of the crystalline MFI structure and a moderate growth in intensity of the most intense diffraction peaks with increasing severity of calcination conditions (Figure S2).^40^

Ar physisorption indicated that all catalysts possessed mixed type I–II isotherms and hysteresis loop type 4, in agreement with the microporous nature of the MFI zeolite framework and the soft mesoporosity induced by intercrystalline particle voids (Figure S3a).^41^ No remarkable variation in the apparent specific surface area (*S*_a_) was observed (Table S1). Similarly, the pore-size distribution was not affected by the calcination treatments, and all catalysts displayed the expected peak at ca. 0.55 nm corresponding to the straight and zigzag channels of the MFI structure (Figure S3b). Therefore, these calcination treatments did not affect the crystalline porous structure of the catalysts.

**Figure 1:**
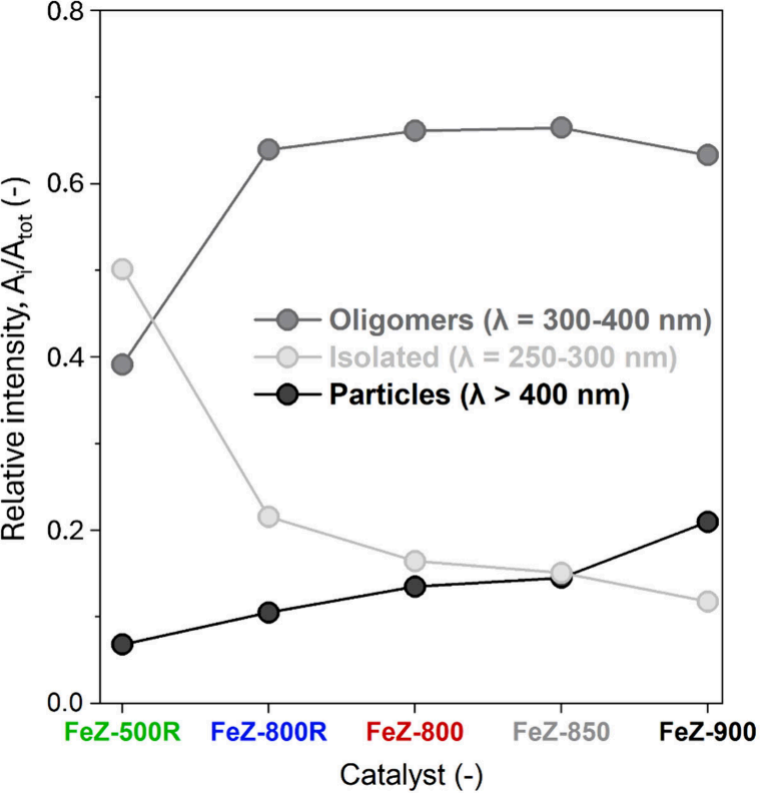
Contribution of isolated, oligomeric, and hematite-like Fe^3+^ species in the dehydrated Fe-ZSM-5 catalysts (Figure S5 and Table S2).

To track the evolution of the Fe species upon calcination, X-ray absorption spectroscopy (XAS), diffuse reflectance ultraviolet–visible spectroscopy (DRUV), and electron paramagnetic resonance (EPR) were employed. The non-phase shift corrected Fourier transformed *k*^2^-weighted EXAFS spectra of the hydrated Fe-ZSM-5 catalysts were dominated by two contributions at ca. 1.5 and 2.7 Å, which are related to the Fe–O and Fe–Fe scattering paths, respectively (Figure S4).^42^ The most notable variation in the spectra was a progressive increase in intensity of the Fe–Fe scattering path in catalysts calcined under more severe calcination conditions, suggesting an increase of the average degree of Fe agglomeration.^26^ This behavior is not sufficient to conclude on the evolution of monomeric/isolated sites because their contribution is dominated by that of the oligomeric species, all structures being averaged by the bulk-sensitive EXAFS technique. On the contrary, UV spectroscopy can provide access to such information in a selective manner. The UV–vis spectrum of Fe-exchanged zeolites exhibits charge transfer transitions (O^2–^ → Fe^3+^) whose energy is a function of the degree of agglomeration (Figure S5).^43^ Semiquantitative determination of the various Fe^3+^ species is possible using Gaussian functions following reported procedures (Table S2).^25^ Four Gaussian bands were required for a proper fit of the DRUV spectra (Figure S5). Bands peaking at ca. 270 nm, in the range of 330–390 nm and at about 450 nm, were found irrespective of sample and are assigned to isolated Fe^3+^ sites, small Fe^3+^ oligomers (Fe_*x*_O_*y*_(OH)_*z*_), and hematite-like particles (Fe_*x*_O_*y*_), respectively.^25,44^ The relative contents of these extra-framework Fe^3+^ species (*A*_*i*_/*A*_tot_) along the calcination coordinate are displayed in Figure 1. By employing more severe calcination conditions, the content of isolated Fe^3+^ sites decreased and was compensated for by a fraction of Fe_*x*_O_*y*_(OH)_*z*_ oligomers and by a monotonic increase in the content of Fe_*x*_O_*y*_-like particles. Moreover, the increase in total DRUV intensity characterizing the spectrum of catalysts following the severity of the calcination treatment (Table S2 and Figure S6) indicates that a growing fraction of Fe^2+^ sites is transformed in DRUV-detectable Fe^3+^ species,^45^ possibly belonging to Fe_*x*_O_*y*_(OH)_*z*_ oligomers and Fe_*x*_O_*y*_-like particles (Figure S6).

**Figure 2:**
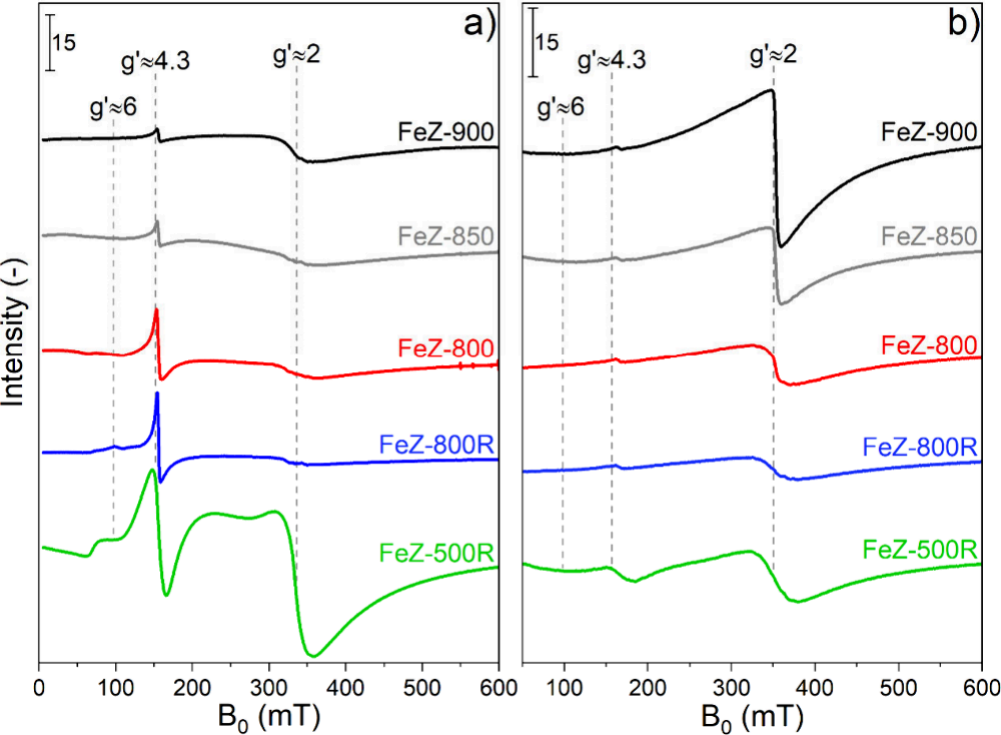
EPR spectra of the dehydrated Fe-ZSM-5 catalysts measured at −243 °C (a) and 25 °C (b). Effective *g*′ factors are indicated by vertical dashed lines.

The electron paramagnetic resonance (EPR) spectra of the dehydrated catalysts recorded at −243 °C are displayed in Figure 2a and were characterized by three signals at effective *g*′ values of *g*′ ≈ 6, *g*′ ≈ 4.3, and *g*′ ≈ 2. Due to their half-integer spin state, only Fe^3+^ sites are EPR-accessible, whereas the integer-spin Fe^2+^ sites remain EPR silent.^46^ The signal at *g*′ ≈ 6 is assigned to isolated high-spin Fe^3+^ sites in β-cationic position (Fe_β_^3+^ in distorted 6-membered rings), possessing a distorted axial symmetry possibly corresponding to a distorted octahedron,^47^ or in planar or square pyramidal environments^19,48^ and being located in the channel intersections of ZSM-5.^49^ The signal at *g*′ = 4.3 corresponds to isolated high-spin Fe^3+^ sites in tetrahedral coordination, based on the rhombicity (E/D = 0.33) of their zero field splitting,^19,50,51^ that are accommodated in γ position (Fe_γ_^3+^), a complex unit of the framework composed of 5- and 6-membered rings in the sinusoidal channels of ZSM-5.^52^ Comparison of the spectra in Figure 2a indicates that the signals associated with Fe_γ_^3+^, and especially to Fe_β_^3+^ sites, became narrower and lost intensity in catalysts calcined under severe calcination conditions, suggesting a loss of these species. The transitions at *g*′ ≈ 2 can arise either from highly symmetric isolated Fe^3+^ sites (paramagnetic) in α position of the sinusoidal channels of ZSM-5^49^ or from Fe^3+^ species in Fe_*x*_O_*y*_(OH)_*z*_ oligomers and in small Fe_*x*_O_*y*_-like clusters (antiferromagnetic) in the main channels of ZSM-5.^51,53^ These species can be discriminated because the intensity (*I*) of paramagnetic species is a function of the temperature according to the Curie–Weiss law: *I* ∼ 1/*T*.^54^ Comparison of the spectra recorded at −243 and 25 °C (Figure 2a,b, respectively) shows that the signal at *g*′ ≈ 2 became broader and less intense in FeZ-500R, while it was narrower and progressively more pronounced in the rest of the Fe-ZSM-5 catalysts. The different temperature dependence indicates that mostly symmetric isolated Fe^3+^ sites contributed to the signal at *g*′ ≈ 2 in FeZ-500R. Differently, a growing content of Fe^3+^ species in Fe_*x*_O_*y*_(OH)_*z*_ oligomers and Fe_*x*_O_*y*_-like clusters characterizes the transition at *g*′ ≈ 2 in the spectra of FeZ-800R, FeZ-800, FeZ-850, and FeZ-900.

Combining the results collected by XAS, DRUV, and EPR, we can conclude that the five Fe-ZSM-5 catalysts experienced a progressive Fe-agglomeration following the severity of the calcination treatment (FeZ-500R < FeZ-800R < FeZ-800 < FeZ-850 < FeZ-900). This process involves isolated (EPR-active) Fe^3+^ sites in γ- and especially in β-cationic position and probably also their (EPR-silent) Fe^2+^ counterparts located in the same cationic environments that agglomerate in (DRUV- and EPR-active) Fe_*x*_O_*y*_(OH)_*z*_ oligomers and Fe_*x*_O_*y*_-like clusters in the main straight channels and/or on the outermost part of the porous structure.

**Figure 3:**
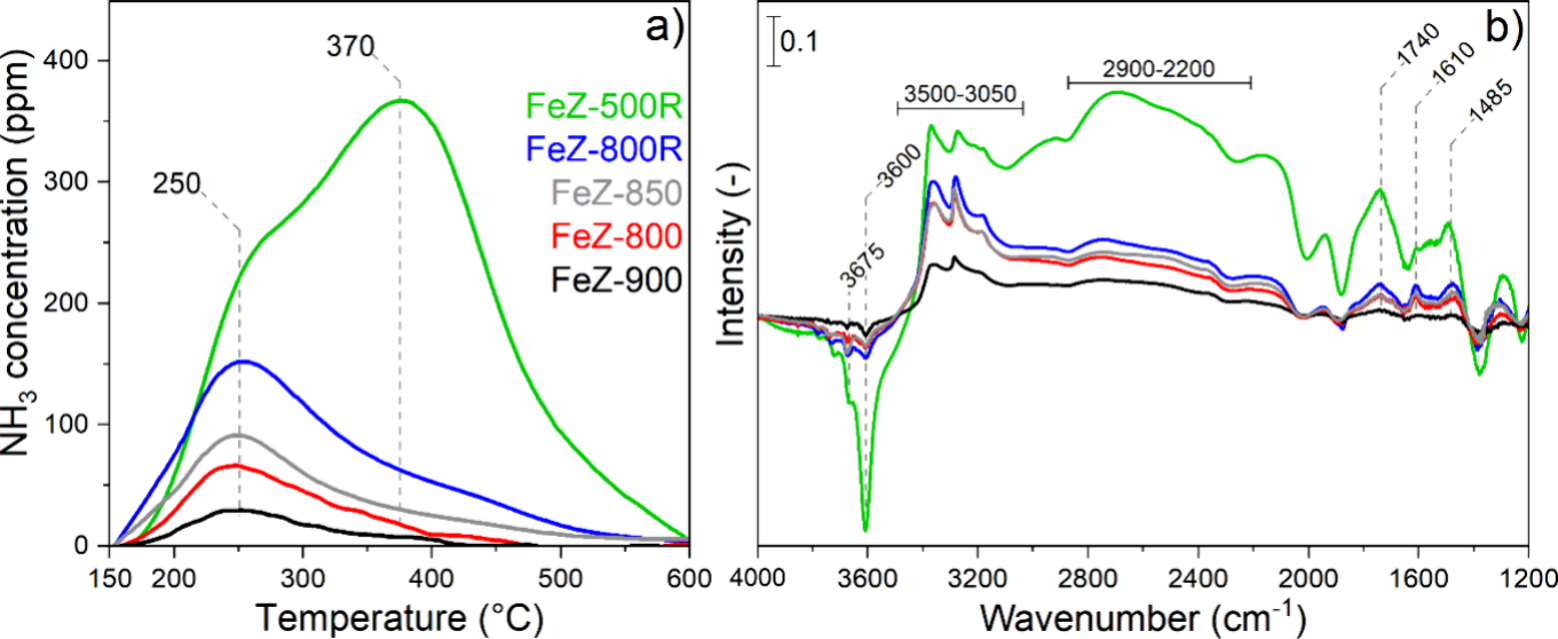
NH_3_-TPD experiments (a) and *in situ* DRIFT spectra (b) recorded after adsorption–desorption of 1000 ppm of NH_3_/Ar over the dehydrated Fe-ZSM-5 catalysts at 150 °C.

NH_3_-TPD experiments were conducted to determine the evolution of the acidic properties of the catalysts. Comparison of the desorption profiles (Figure 3a) indicates a remarkable loss of NH_3_ storage capacity (Table S1, NH_3,SC_), especially in the high-temperature region that is assigned to NH_3_ coordinated to the Brønsted acid sites (BAS, ca. 370 °C).^55,56^ In agreement with the results concerning the agglomeration of the Fe species, the severe calcination conditions probably also induced dislodgment of framework-Al atoms to extra-framework positions, ultimately arising in the loss of BAS.^26^

Adsorption on acid sites was also characterized using the *in situ* DRIFT spectra collected after exposure to 1000 ppm of NH_3_/Ar and flushing with Ar at 150 °C (Figure 3b). The negative peaks at 3675 and 3600 cm^–1^ are assigned to the hydroxyl group of extra-framework Fe^3+^ sites and BAS, respectively,^57^ to which NH_3_ binds. The corresponding vibrational modes of NH_4_^+^ adsorbed on BAS were found between 2900 and 2200 cm^–1^ (ν_N–H_), at 1740 cm^–1^ (δ_N–H,sym_), and at 1485 cm^–1^ (δ_N–H,asym_).^56,58^ Peaks related to molecular NH_3_ bound to Lewis acid sites (LAS) in the range of 3500–3050 cm^–1^ (ν_N–H_) and at 1610 cm^–1^ (δ_N–H_) appeared in the catalysts calcined above 500 °C. The overall intensity of the peaks below 3500 cm^–1^ decreased with increasing severity of the calcination treatment and was mirrored by smaller negative signals above 3500 cm^–1^, thus confirming the agglomeration of isolated Fe^3+^ species and the loss of BAS.

### Catalytic Activity and Reaction Rates

**Figure 4:**
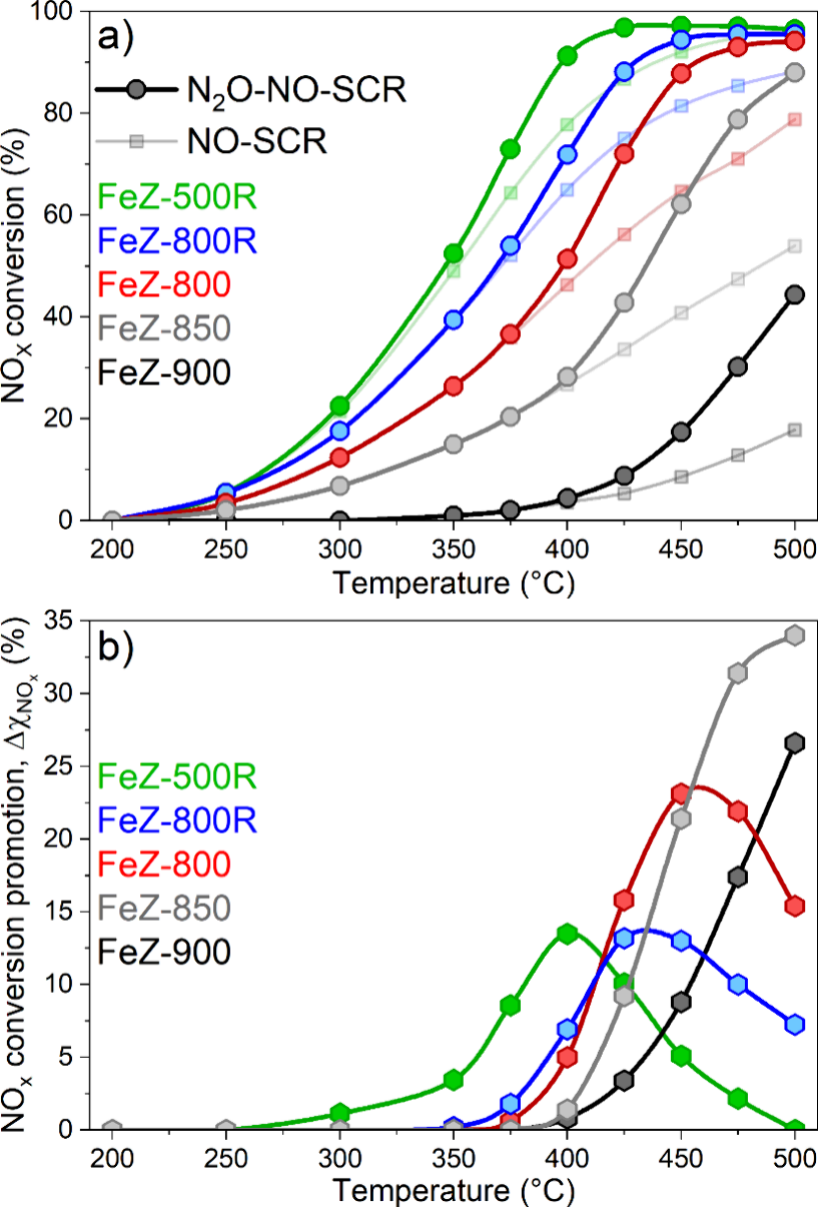
NO_*x*_ conversion under N_2_O-NO-SCR and NO-SCR conditions (a) and corresponding promotion of the NO_*x*_ conversion (Δχ_NOx_) (b) in the presence of N_2_O.

In order to investigate the effect of N_2_O on the conversion of NO, the catalysts were tested in the absence (NO-SCR) and presence of 500 ppm of N_2_O in the feed (N_2_O-NO-SCR, Figure 4a) using an excess of NH_3_ corresponding to [NH_3_]/[NO_*x*_] = 1.2. Despite the feed containing N_2_O and NO, the conversions are expressed in terms of N_2_O and NO_*x*_ to account for the possible appearance of NO_2_. The NO_*x*_ conversion during NO-SCR decreased, following the severity of the calcination treatment. This is primarily associated with the increase in the degree of Fe agglomeration, causing a decrease in the fraction of Fe atoms possibly participating in the reaction as well as the content of most active Fe sites.^59^ In light of the nonproportional loss of acidity and BAS, these two parameters cannot solely explain the NO_*x*_ conversion trends.

Irrespective of catalyst, the addition of 500 ppm of N_2_O (N_2_O-NO-SCR) promoted the NO_*x*_ conversion (Figure 4a). This is in agreement with the results obtained with an independent catalyst based on Fe-FER^19^ and demonstrates that the beneficial role of N_2_O on the conversion of NO can manifest also over MFI-based Fe-exchanged zeolite catalysts. Simultaneously, both the extent of promotion at a given temperature (Δχ_NO_*x*__) and the temperature at which the onset of promotion occurred were catalyst dependent. In order to strengthen these observations, Δχ_NO_*x*__ is plotted as a function of reaction temperature in Figure 4b. Below 400 °C, the onset temperature shifted progressively to higher values, and Δχ_NO_*x*__ decreased in catalysts calcined under more severe conditions. Differently, above 400 °C, this latter trend started reversing, and the catalysts calcined under more severe calcination conditions gradually exhibited higher Δχ_NO_*x*__. The comparison between the NO_*x*_ conversion under N_2_O-NO-SCR conditions (Figure 4a) and Δχ_NO_*x*__ (Figure 4b) suggests that this reversed regime occurred because full NO_*x*_ conversion was progressively attained by FeZ-500R, FeZ-800R, and FeZ-800. Indeed, the Δχ_NO_*x*__ curves in these three catalysts began to decline above 400, 425, and 450 °C (Figure 4b), respectively, coinciding with ca. 90% NO_*x*_ conversion under N_2_O-NO-SCR conditions (Figure 4a). Thus, above 400 °C, full NO_*x*_ conversion progressively limits the effect of N_2_O on the NO-SCR reaction as evidenced by the maximum value of Δχ_NO_*x*__.

In the case of N_2_O conversion under N_2_O-NO-SCR conditions (Figure S7f–l and Table 1), the onset of the beneficial effect of N_2_O on the NO-SCR reaction coincided with the onset of the N_2_O conversion, as observed before.^19^ Moreover, although all catalysts showed low or negligible conversion in the direct N_2_O decomposition, the onset of the NO mediated-N_2_O decomposition reaction also seemed to coincide with the promotion of the NO_*x*_ conversion under N_2_O-NO-SCR conditions. This agrees with the proposal that a N_2_O-assisted NO oxidation step is crucial for the N_2_O-NO-SCR reaction.^13,19^ NO_2_ (2 ppm) was detected only at 400 °C on Fe-500R, in agreement with the mechanistic implication of the use of excess NH_3_ proposed recently.^19^ While further detailed study should be performed to assess the effect of acidity, we did not find any correlation in this series of samples between acidity and activity toward N_2_O-NO-SCR.

**Figure 5:**
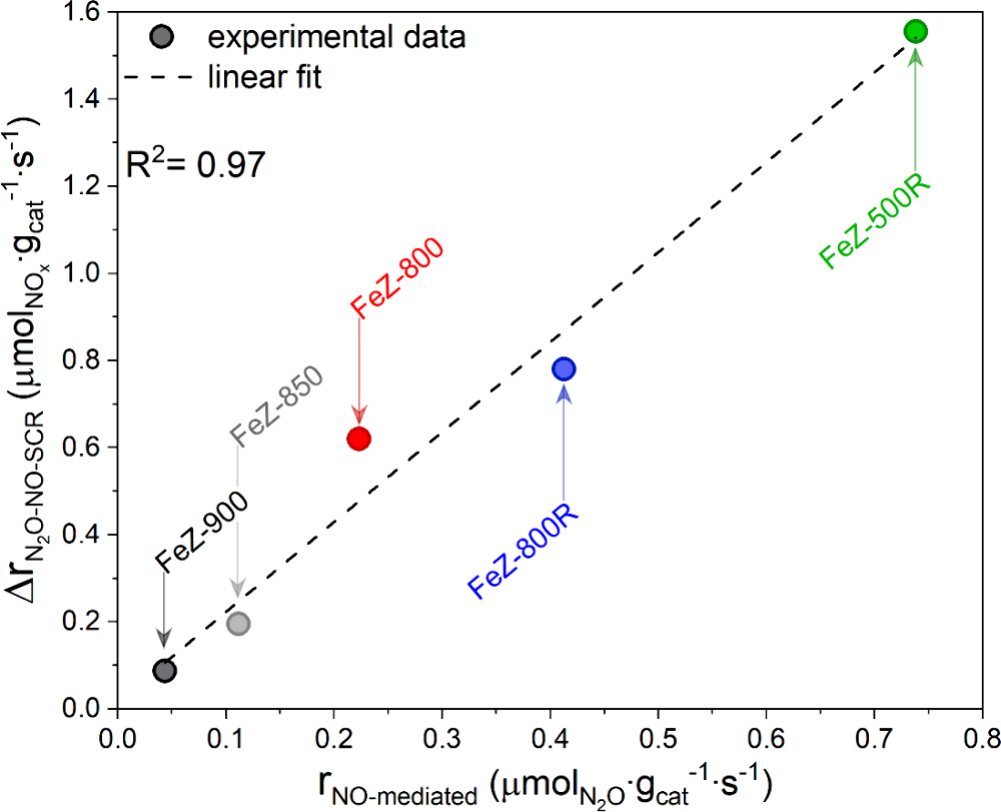
Relationship between the promotion of the NO-SCR reaction rate in the presence of N_2_O (Δ*r*_N_2_O-NO-SCR_) and the reaction rate for N_2_O conversion under NO-mediated N_2_O decomposition conditions (*r*_NO-mediated_) at 400 °C.

To investigate a possible relationship from a kinetic perspective, we calculated and compared the normalized reaction rates for N_2_O conversion under NO mediated-N_2_O decomposition (*r*_NO-mediated_) and for NO_*x*_ conversion in the presence of N_2_O (Δ*r*_N_2_O-NO-SCR_) at 400 °C (see Experimental Section) in Figure 5. A comparison between the catalysts was possible only at this temperature because at 400 °C all catalysts displayed an extent of Δχ_NO_*x*__ and the effect of N_2_O on the NO-SCR reaction was limited above 400 °C (Figure 4b). The analysis of the reaction rates of these catalytic data (Figure 5) indicates that *r*_NO-mediated_ and Δ*r*_N_2_O-NO-SCR_ are proportional under the experimental conditions in which the NO_*x*_ conversion did not reach 100%. Further confirmation of this finding was obtained by the plots of *r*_NO-mediated_ and Δ*r*_N_2_O-NO-SCR_ for each catalyst as a function of the reaction temperature, neglecting the data in which limitation by full NO_*x*_ conversion was observed (Figure S8). Linear functions appropriately fit the experimental data in all catalysts and within the whole temperature regime taken into consideration.

These results unambiguously demonstrate the crucial role of the N_2_O-assisted NO oxidation in the N_2_O-NO-SCR reaction, which we proposed recently studying a commercial Fe-FER catalyst using various spectroscopic methods.^19^ Furthermore, the simultaneous loss of monomeric Fe sites and of catalytic activity in both reactions suggests a crucial role of isolated Fe species in governing N_2_O-assisted NO oxidation and consequently the enhanced NO conversion under N_2_O-NO-SCR conditions.

### Spectroscopic Analysis

The kinetic analysis indicated that the activity of the catalysts in the NO-mediated N_2_O decomposition reaction determines the promotion of NO conversion under the N_2_O-NO-SCR conditions. To achieve molecular insights into the behavior of relevant species involved in the reaction, dynamic *operando* DRIFTS and XAS experiments were conducted according to the modulated excitation (ME) approach.

**Figure 6:**
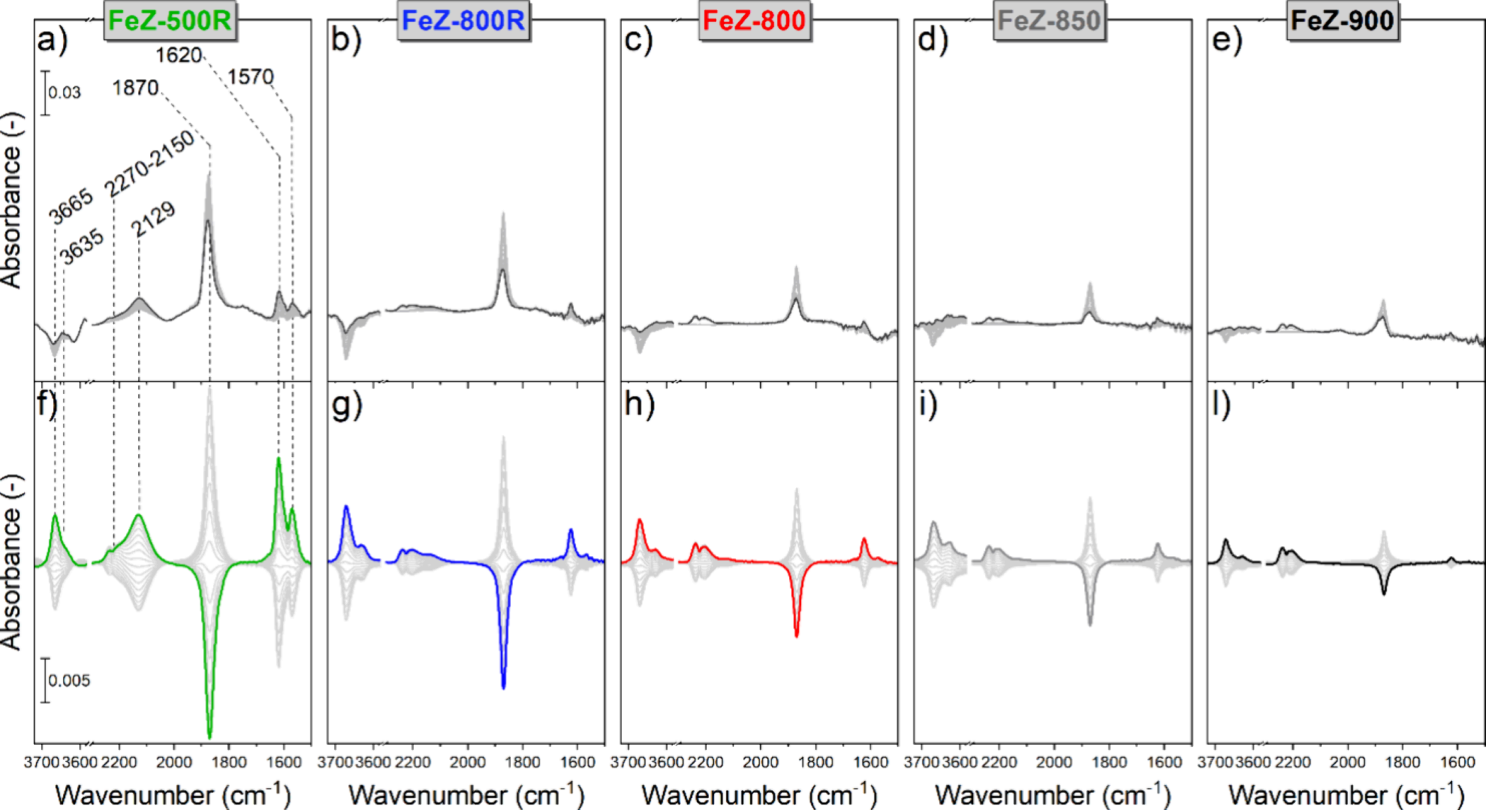
Averaged time-resolved (a–e) and phase-resolved (f–l) *operando* DRIFT spectra of the Fe-ZSM-5 catalysts during 4 min pulses of 1000 ppm of N_2_O/Ar in a flow of 1000 ppm of NO/Ar at 400 °C. The bold spectra in (a–e) are drawn to indicate the effect of N_2_O addition on the DRIFT spectra of the catalysts equilibrated under NO. The bold spectra in (f–l) are drawn to guide the eye (φ^PSD^ = 120°).

*Operando* DRIFTS was performed under conditions of NO-mediated N_2_O decomposition. Repeated pulses of N_2_O over the catalysts equilibrated in NO feed caused the reversible perturbation of several vibrational modes above 3600 cm^–1^, in the 2300–2100 cm^–1^ region, and below 1900 cm^–1^ in all catalysts (Figure 6). The negative bands at 3665 and 3635 cm^–1^ are assigned to Fe–OH sites (ν_O–H_).^57^ The signal of NO^+^ probably adsorbed on BAS (ν_N–O_, 2129 cm^–1^)^60^ was clearly visible only on FeZ-500R and to a much lesser extent on FeZ-800R and on the other catalysts. Finally, the bands below 2000 cm^–1^ are associated with mononitrosyl species adsorbed on isolated Fe^2+^ sites (Fe_γ_) or on (FeO)_*n*_ clusters (ν_N–O_, 1870 cm^–1^) and with a combination of nitro (NO_2_, 1620 cm^–1^) and nitrate (NO_3_, 1570 cm^–1^) species coordinated to Fe.^57,60−62^ According to the progressive loss of intensity of the Fe–NO band with calcination (Figure 6a–e) and of isolated Fe species detected by XAS, DRUV, and EPR spectroscopy (Figure S4, Figure 1, and Figure 2), the signal at 1870 cm^–1^ (ν_N–O_) is assigned to NO adsorption on isolated Fe^2+^ sites in γ-position.^62^

The time-resolved data (Figure 6a–e) indicated that N_2_O introduction to all catalysts promoted partial loss of the signal associated with Fe^2+^–NO and the increase in intensity of the features related to Fe–OH, BAS–NO^+^, Fe–NO_2_ and Fe–NO_3_. The corresponding phase-resolved data sets (Figure 6f–l) showed that (i) the spectroscopic response was qualitatively similar among the catalysts and (ii), except for Fe^2+^–NO, all other bands were in phase with the signals of gas-phase N_2_O (ν_N–N_, 2270–2150 cm^–1^). Therefore, N_2_O introduction promoted the formation of Fe–OH sites and the consumption of Fe^2+^–NO by means of an oxidation process producing Fe–NO_2_, BAS–NO^+^, and Fe–NO_3_ species. This is in agreement with the occurrence of the NO-mediated N_2_O decomposition reaction.^19,63^ The extent of the spectroscopic response delivered by PSD (Figure 6f–l) was significantly lower in catalysts calcined under more severe conditions, thus possessing a lower fraction of isolated Fe sites, indicative of progressively poorer activity in catalyzing the oxidative transformation of Fe^2+^–NO. The decreased reactivity in the NO-mediated N_2_O decomposition reaction monitored in these *operando* experiments (Figure S9) and in previous catalytic tests (Figure S7f–l) confirms this hypothesis.

The increased intensity in the Fe–OH bands (3665 and 3635 cm^–1^, Figure 6f–l) upon N_2_O addition can be related to the formation of novel Fe^3+^–OH moieties once N_2_O is activated over specific isolated square planar Fe^2+^ centers in β-cationic positions in the form of square pyramidal Fe^3+^–OH sites.^19,64^ However, reference NO modulation experiments (Figure S10) indicated that also NO adsorption influences the same Fe–OH bands. Precisely, the opposite phase relationship between the bands associated with Fe–OH and Fe^2+^–NO revealed that NO causes a reversible process in which some OH– groups bound to Fe centers are consumed or formed during its adsorption or desorption, respectively. This could be explained by the formation of Fe–O(H)–NO species damping the stretching vibration of O–H bonds.^10^

To determine whether also N_2_O activation in the form of Fe^3+^–OH sites contributes to the intensification of the signals at 3665 and 3635 cm^–1^, the ratio between the maximum amplitude variation obtained for the Fe^2+^–NO and Fe–OH signals by PSD analysis (*R*_NO/OH_) was compared between the NO- and N_2_O-modulation experiments (Figure S10 and Table S3). While the *R*_NO/OH_ ratios were different among the catalysts, the one calculated for the NO pulse experiments was systematically higher than the values obtained in the N_2_O modulation experiments. Thus, the freeing of the Fe sites upon NO consumption does not entirely explain the increased intensity of the ν_O–H_ modes in Fe–OH sites during N_2_O modulation experiments in NO, suggesting that N_2_O activation in the form of Fe^3+^–OH sites also contributes to the vibrational modes at 3665 and ca. 3635 cm^–1^. Accordingly, these spectroscopic features were detected upon addition of N_2_O over the dehydrated FeZ-500R catalyst (Figure S11), confirming its activation in the form of Fe^3+^–OH sites. Therefore, the intensification of the Fe–OH signals upon N_2_O addition arises from a combination of the reaction processes (N_2_O activation) and adsorption/desorption (NO coordination).

FeZ-500R, FeZ-800R, and FeZ-800 were also studied by *operando* XAS by exposing them to repeated pulses of N_2_O in a constant feed of NO-SCR (Figure S12). The time-resolved data (Figure S12a,c) showed that N_2_O introduction produced only a subtle shift of the absorption edge to higher energy, with an extent that was dependent on the catalyst. The phase-resolved spectra (Figure S12d–f) revealed that the changes around the absorption edge corresponded to the loss of intensity in two contributions at 7121 and 7125 eV and that two pre-edge features (7112/7115) were also reversibly affected by the N_2_O pulses. The pre-edge components at 7112 and 7115 eV characterize 1s → 3d electronic transitions in Fe^2+^ and Fe^3+^ ions, respectively.^65^ Thus, the symmetric evolution of the pre-edge features indicated that a fraction of active Fe^2+^ ions was reversibly consumed and transformed in their Fe^3+^ counterparts in the presence of N_2_O. In analogy to the *operando* DRIFTS results, the spectra of FeZ-800R and FeZ-800 displayed a lower spectroscopic response to the N_2_O pulses, suggesting that the content of reactive Fe^2+^ species and of related Fe^2+^/Fe^3+^ dynamic decreased with the increasing extent of agglomeration of the Fe species. The corresponding online MS data (Figure S12g–i) showed that N_2_O addition favored NO conversion, thus proving that the Fe^2+^/Fe^3+^ redox pairs are related to the occurrence of the N_2_O-NO-SCR reaction. However, the extent of promotion of the NO conversion decreased as the Fe agglomeration increased, in agreement with the catalytic results (Figure 4b), suggesting that the lower concentration of redox active Fe^2+^ sites is responsible for the decrease of the NO conversion under N_2_O-NO-SCR conditions.

### Structure–Activity Relationships and Active Sites

The kinetic analysis (Figure 5 and Figure S8) indicated that the promotion of the NO conversion under N_2_O-NO-SCR conditions is governed by the activity of the catalyst in the NO-mediated N_2_O decomposition reaction. Analysis of this latter process by *operando* DRIFTS (Figure 6) revealed that the catalysts calcined under more severe conditions contained less isolated Fe species and suffered from decreased activity in catalyzing the oxidative transformation of Fe^2+^–NO. On the one hand, this behavior is likely related to the lower surface concentration of Fe^2+^–NO as a result of the decreased content of isolated Fe sites (Figure 6a–e). This is in agreement with the observation that, in the NO-mediated N_2_O decomposition, NO reacts after coordinating to Fe.^8^ On the other hand, the time-resolved spectra also indicated that Fe^2+^–NO was not entirely consumed at the end of the N_2_O pulses (Figure 6a–e), thus suggesting that the low surface concentration of adsorbed NO might not be the only parameter controlling the NO-mediated N_2_O decomposition reaction and, consequently, also the NO_*x*_ conversion under N_2_O-NO-SCR conditions.

We recently demonstrated^19^ that N_2_O requires an activation step before it can oxidize adsorbed NO.^8^ Thus, this preliminary step might also control the NO-mediated N_2_O decomposition activity. In principle, one could monitor the ability of the catalyst to activate N_2_O from the intensity of the Fe–OH signals in the time-resolved DRIFTS data (3665 and 3635 cm^–1^, Figure 6a–e). However, quantifying the N_2_O activation from these DRIFTS experiments is challenging (i) because the ν_O–H_ modes in activated Fe^3+^–OH sites and in Fe–OH species restored by consumption of NO overlap (Figure S10) and (ii) because the *R*_NO/OH_ values are catalyst dependent (Table S3).

To overcome these challenges, we performed dedicated experiments to quantify the concentration of the Fe sites able to activate N_2_O (α-sites, *C*_α_) and the concentration of the Fe sites able to coordinate NO (*C*_Fe-NO_).

**Figure 7:**
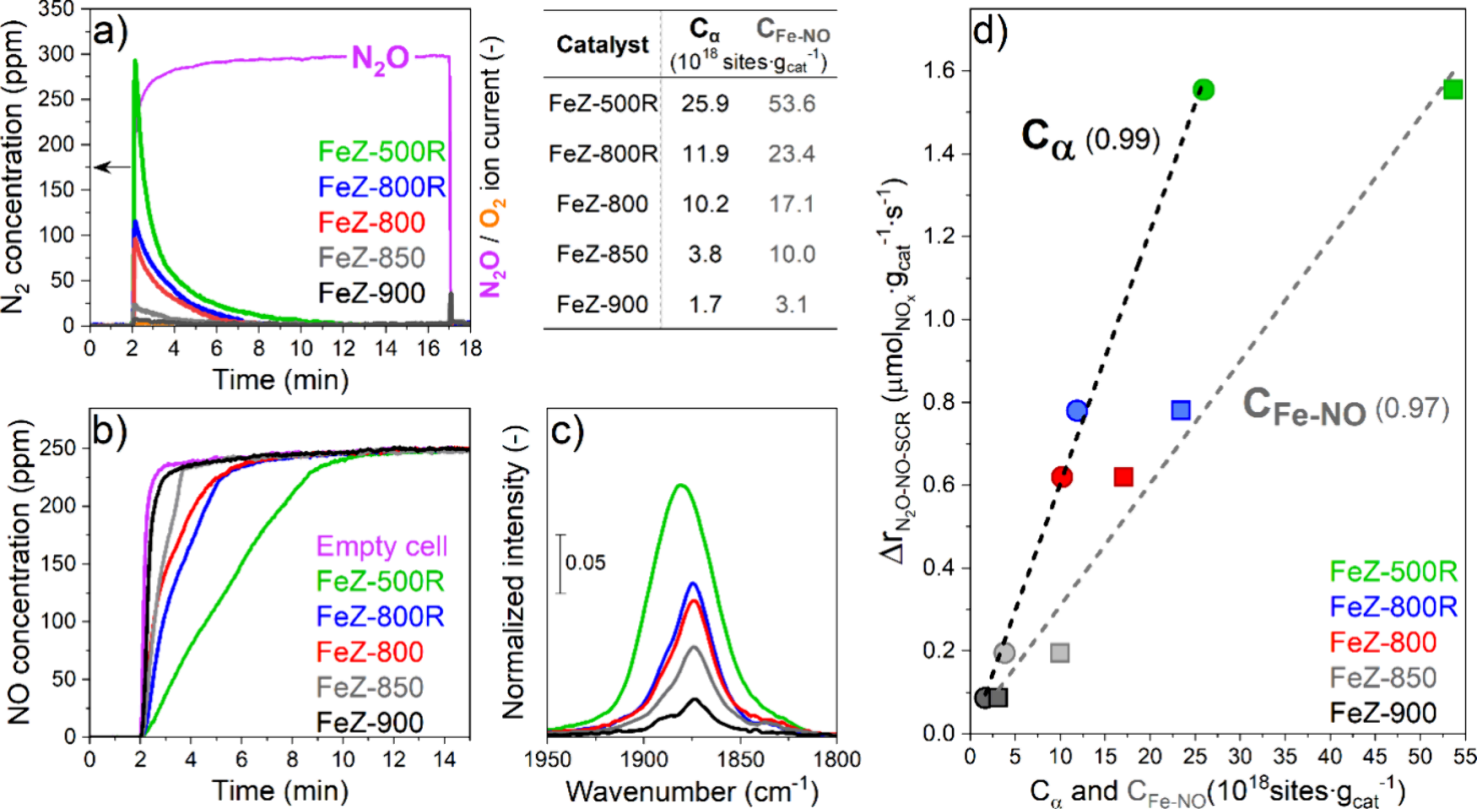
N_2_ concentration (a) during N_2_O addition experiments at 200 °C. NO concentration (b) and *in situ* DRIFT spectra (c) during NO saturation experiments at 200 °C. Relationship between the promotion of the NO-SCR reaction rate in the presence of N_2_O (Δ*r*_N_2_O-NO-SCR_) at 400 °C and the concentrations of α-sites (*C*_α_) and of the Fe sites able to adsorb NO (*C*_Fe-NO_) (d). The table shows the *C*_α_ and *C*_Fe-NO_ values for each catalyst. Dashed lines in (d) are linear fits and numbers in brackets represent their *R*^2^.

*C*_α_ was determined by N_2_O addition experiments at 200 °C (Figure 7a).^39^ The introduction of N_2_O caused a transient N_2_ production in the online MS (*m*/*z* = 28), in agreement with N_2_O activation via N_2_–O bond cleavage and consequent saturation of the α-sites in form of (α-O) (eq 1). The O_2_ signal (*m*/*z* = 32) maintained background levels in each experiment, confirming the validity of the experimental approach. Although all catalysts were able to activate N_2_O, the extent of N_2_ production and thus the value of *C*_α_ calculated according to eq 7 decreased significantly with increasing the severity of calcination conditions and, consequently, with increasing the degree of Fe agglomeration (see table in Figure 7).

For *C*_Fe-NO_, we conducted NO saturation experiments at 200 °C (Figure 7b,c). NO adsorption was followed until saturation of the corresponding MS signal (*m*/*z* = 30, Figure 7b) and by the appearance of an absorption peak at ca. 1875 cm^–1^ (Fe^2+^–NO)^62^ in the DRIFT spectrum (Figure 7c). Following the severity of the calcination treatment, saturation of the NO concentration occurred more rapidly (Figure 7b), in agreement with the less intense Fe^2+^–NO signal recorded simultaneously by *in situ* DRIFTS (Figure 7c). Therefore, as quantified through the *C*_Fe-NO_ parameter by repeating the NO saturation experiment with the empty spectroscopic cell (see the table in Figure 7), the catalysts calcined under harsher conditions displayed remarkable loss of NO adsorption properties, in agreement with the time-resolved *operando* DRIFT spectra collected during NO-mediated N_2_O decomposition (Figure 6a–e).

These results demonstrate that the loss of isolated Fe sites has a negative impact on the concentrations of the Fe sites involved in N_2_O activation and in NO adsorption. Because the *operando* DRIFTS experiments (Figure 6a–e) indicated that the loss of isolated Fe sites also caused decreased NO oxidation activity, we propose that the loss of N_2_O activation (*C*_α_) and of NO adsorption (*C*_Fe-NO_) properties under severe calcination conditions determine reduced activity in the oxidative transformation of Fe^2+^–NO.

In addition to the NO-mediated N_2_O decomposition, the oxidation of Fe^2+^–NO is an essential step also during N_2_O-NO-SCR.^19^ In this recent study, we proposed that N_2_O favors NO conversion by promoting the oxidation of NO to an oxidized intermediate, which yields N_2_ and water products upon reaction with NH_3_. Therefore, *C*_α_ and *C*_Fe-NO_ could also directly influence the promotion of the NO conversion during N_2_O-NO-SCR. This hypothesis was confirmed by the direct relationship between these two catalyst parameters and the increase in the normalized reaction rate for NO_*x*_ conversion in the presence of N_2_O at 400 °C (Δ*r*_N_2_O-NO-SCR_, Figure 7d), which unequivocally demonstrates that Fe sites involved in processes of N_2_O activation and NO adsorption govern the promotion of the NO_*x*_ conversion under N_2_O-NO-SCR reaction conditions.

Two observations help us to conclude about the nature of the active sites. DRUV and EPR spectroscopy (Figure S5, Figure 1 and Figure 2, respectively) suggested that the thermal treatments caused the transformation of isolated Fe^2+^ sites in γ- and β-cationic positions into Fe^3+^ likely located in Fe_*x*_O_*y*_(OH)_*z*_ oligomers and in Fe_*x*_O_*y*_-like clusters. Furthermore, considering that (i) EPR spectroscopy (Figure 2a) showed a higher stability of isolated Fe species in the γ-cationic position and that (ii) the values of *C*_Fe-NO_ were systematically higher than those of *C*_α_ (table in Figure 7), we conclude that the Fe sites involved in N_2_O activation and NO adsorption are isolated Fe^2+^ centers in β-^5^ and γ-cationic^66^ positions, respectively. This structure–activity relationship agrees with the lower extent of redox dynamics observed in the *operando* XAS experiments (Figure S12) that is crucial for the N_2_O-NO-SCR activity.^19^

It is worth noticing that N_2_O activation resulted in an individual, very resolved, Fe^3+^–OH band (3649 cm^–1^) in Fe-FER,^19^ while two vibrational modes (3665 and ca. 3635 cm^–1^) appeared in this Fe-ZSM-5 series (Figure 6). The difference is likely related to the different framework structure between FER and ZSM-5 and particularly to their different ability to accommodate isolated square planar Fe^2+^ centers in β-cationic positions (6-membered rings).^49^ FER ensures a pair of identical β-cationic positions in each of its cavities (i.e., the connection between the various straight channels). Differently, in ZSM-5, two different deformed six-membered rings can be found at the intersection between straight and sinusoidal channels.^67^ Therefore, we propose that the signals at 3665 and ca. 3635 cm^–1^ belong to slightly different isolated square planar Fe^2+^ centers in both six-membered structures within the ZSM-5 framework, which can activate N_2_O and form Fe^3+^–OH moieties.

In summary, since severe calcination conditions transform some of the catalytically relevant mononuclear Fe^2+^ species in β- and γ-cationic positions into inactive large agglomerates, the N_2_O activation, the NO adsorption capacity, the fraction of redox active species, and the ability of the catalyst to promote the conversion of NO upon addition of N_2_O under N_2_O-NO-SCR conditions decrease. The role of changes in acidity due to calcination at higher temperature remains elusive, likely because N_2_O activation and NO adsorption require predominantly Fe centers, and the effects on Fe speciation dominate.

The results reported in this study unequivocally prove the involvement of Fe-mediated N_2_O activation and NO adsorption (and oxidation) during the simultaneous conversion of N_2_O and NO under N_2_O-NO-SCR conditions. These processes are catalyzed by a minority of isolated Fe^2+^ sites occupying β- and γ-cationic exchange positions in the straight channels and at the intersection between straight and sinusoidal channels of the ZSM-5 framework and possess distorted square-planar and tetrahedral coordination, respectively. Maximization of the amount of these centers is therefore essential to exploit to a greater extent the promotion of the NO conversion under nonlimiting conditions, to attain this effect at lower reaction temperature, and thus to optimize the reaction performance during N_2_O-NO-SCR.

## Conclusions

In this study, the N_2_O-NO-SCR reaction was investigated over five Fe-ZSM-5 catalysts possessing the same Fe loading but different degrees of Fe dispersion and acidity, by combining results from catalytic experiments, *ex situ* characterization, and *in situ/operando* spectroscopies. Catalytic results showed that at a high degree of Fe dispersion (i) the temperature at which N_2_O manifests promotion of the NO_*x*_ conversion decreases and (ii) the extent of promotion of the NO_*x*_ conversion at the same temperature increases. By correlating the normalized reaction rates, it was observed that the promotion of the NO_*x*_ conversion is governed by the activity of the catalysts in the NO-mediated N_2_O decomposition reaction. This information confirms that the oxidative transformation of NO induced by N_2_O is a fundamental step during the N_2_O-NO-SCR reaction. *Operando* DRIFTS characterization of the NO-mediated N_2_O decomposition also indicated that, the lower the degree of Fe dispersion, the lower is the catalyst activity in promoting the oxidation of NO from the adsorbed state. The catalysts lost oxidation activity because the concentration of Fe atoms able to perform N_2_O activation and NO adsorption decreased with an increasing agglomeration of Fe, thus proving the involvement of monomeric Fe sites in these processes as well as the role of these elementary steps in the N_2_O-NO-SCR reaction. In agreement, direct linear functions fit the extent of promotion of the NO_*x*_ conversion triggered by N_2_O under nonlimiting conditions with the concentration of Fe atoms involved in N_2_O activation and in NO adsorption, respectively. We propose that the Fe sites involved in processes of N_2_O activation and NO adsorption (and oxidation) in Fe-ZSM-5 are monomeric Fe^2+^ centers located in β- and γ-cationic exchange positions, respectively. Because we observed very similar behavior with an independent technical Fe-FER catalyst, we believe that our observations can be generalized to Fe-exchange zeolites.

Therefore, the N_2_O-NO-SCR reaction requires catalysts possessing homogeneous distribution of two types of extra-framework Fe^2+^ sites, i.e., in both β- and γ-cationic exchange positions, thereby ensuring simultaneous N_2_O activation and NO oxidation processes, respectively. By optimization of the concentration of these Fe^2+^ centers, N_2_O can manifest its positive influence on the conversion of NO at low temperatures and to a greater extent. Our results indicate that the speciation and location of the Fe cations within the ZSM-5 framework are critical parameters that need to be controlled to achieve an optimized simultaneous conversion of N_2_O and NO.

## Data Availability

All data that support the plots within this manuscript and other findings of this study are available from the corresponding author upon request.

## Supporting Information Available

Experimental characterization details (XRD, Ar-physisorption, EXAFS, DRUV); N_2_O conversion activity; reaction rate correlation; phase-resolved DRIFT spectra of NO modulation; *in situ* DRIFT spectra during N_2_O addition; time- and phase-resolved *operando* XAS spectra (PDF)
