# Supplementary material for: N2O Activation and NO Adsorption Control the Simultaneous Conversion of N2O and NO Using NH3 over Fe-ZSM-5
Source: J Am Chem Soc. 2025 Feb 28;147(10):8978–90. doi: 10.1021/jacs.5c01100 (PMC11912322; doi:10.1021/jacs.5c01100)
Supplement: Supplementary file 1 — ja5c01100_si_001.pdf [file ja5c01100_si_001.pdf]

Supporting Information

N_2O activation and NO adsorption control the simultaneous conversion of N_2O and NO using NH_3 over Fe-ZSM-5

Filippo Buttignol[a,b], Alberto Garbujo[c], Pierdomenico Biasi[c], Oliver Kröcher[a,b] and Davide Ferri[a,*]

[a] Paul Scherrer Institute, PSI Center for Energy and Environmental Sciences, CH-5232 Villigen, Switzerland

[b] Institute for Chemical Sciences and Engineering, École polytechnique fédérale de Lausanne (EPFL), CH-1015 Lausanne, Switzerland

[c] Basic Research Department, Casale SA, CH-6900 Lugano, Switzerland

* Corresponding author: davide.ferri@psi.ch

Table of contents

Supplementary Tables……………………………………………………………………2

Supplementary Figures…………………………………………………………………...5

Supplementary References……………………………………………………………...17

Table S1. Apparent specific surface area (S_a) and NH_3 storage capacity of the catalysts.

**sample**	**S_a** [a)] ($m^2 \cdot g^{-1}$)	**NH_3 storage capacity** [b)] ($mmol \cdot g^{-1}$)
FeZ-500R	343.4	0.872
FeZ-800R	356.1	0.296
FeZ-800	334.5	0.104
FeZ-850	344.7	0.160
FeZ-900	319.9	0.041

[a)] Calculated according to the BET method

[b)] Calculated from integration of the NH_3 desorption peak during the temperature ramp of the NH_3-TPD experiments.

Table S2. Relative content and assignment of Fe species derived from deconvolution of the UV-Vis spectra in Figure S5.

**sample**	**isolated**	**oligomers**	**particles**	**Integrated DRUV intensity**
	λ = 250-300 nm	λ = 300-400 nm	λ > 400 nm	200 nm ≤ λ ≤ 800 nm
	A_i (%)[a)]	A_i (%)[a)]	A_i (%)[a)]	
FeZ-500R	52	41	7	100.4
FeZ-800R	22	67	11	146.9
FeZ-800	17	69	14	152.4
FeZ-850	16	69	15	154.0
FeZ-900	12	66	22	159.4

[a)] A_i/A_{tot}, see Experimental section for details.

Table S3. Maximum amplitude variation of the Fe^{2+}-NO and Fe-OH signals derived from PSD analysis of the dynamic *operando* DRIFT spectra during NO modulation experiments in Ar and N_2O modulation experiments in NO (Figure S10).

sample	NO modulation in Ar			N_2O modulation in NO		
	**Fe^{2+}-NO** (1870 cm^{-1})	**Fe-OH** (3665 cm^{-1})	**$R_{NO/OH}$** [a)]	**Fe^{2+}-NO** (1870 cm^{-1})	**Fe-OH** (3665 cm^{-1})	**$R_{NO/OH}$** [a)]
FeZ-500R	0.052	0.0095	5.47	0.020	0.0054	3.70
FeZ-800R	0.027	0.0067	4.03	0.014	0.0065	2.15
FeZ-800	0.022	0.0042	5.23	0.0084	0.0050	1.68
FeZ-850	0.018	0.0039	4.61	0.0071	0.0045	1.58
FeZ-900	0.010	0.0019	5.26	0.0026	0.0036	0.72

[a)] Ratio between the maximum amplitude variations of the Fe^{2+}-NO and Fe-OH signals

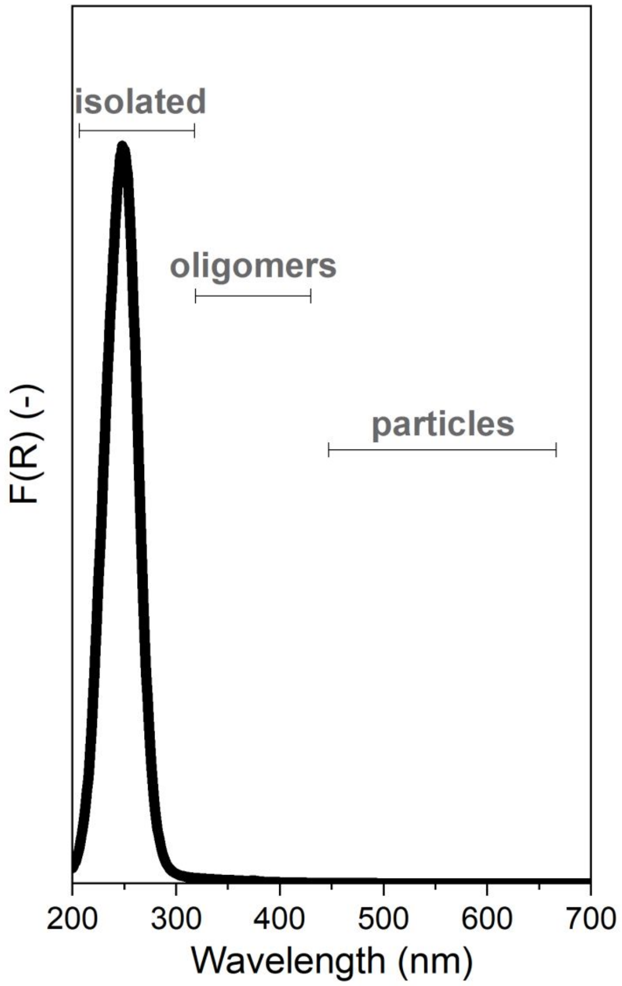

Figure S1. DRUV spectrum of the parent Fe-ZSM-5 material before calcination.[1]

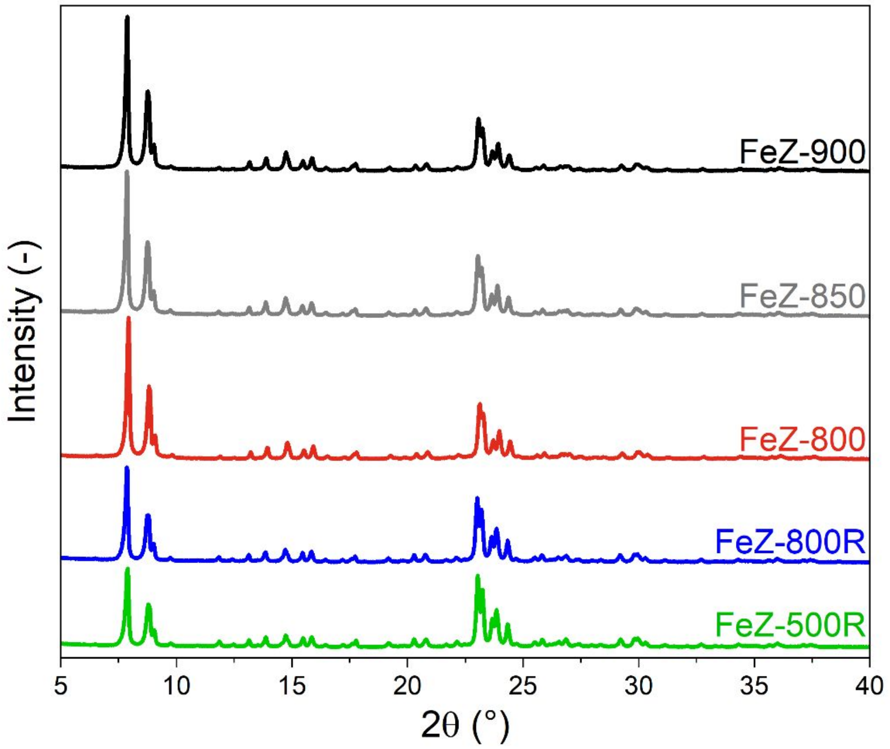

Figure S2. XRD patterns of the Fe-ZSM-5 catalysts.

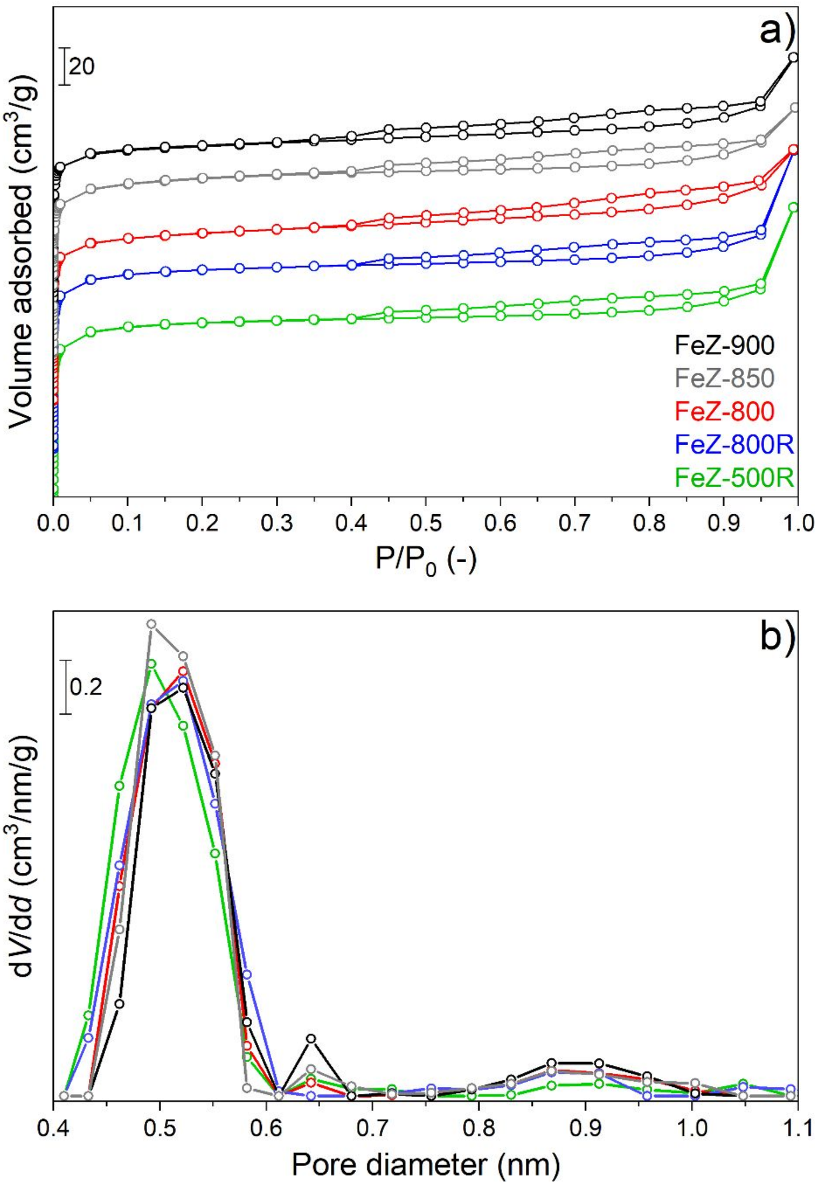

Figure S3. Ar adsorption-desorption isotherms measured at -186 °C (a) and pore-size distribution (b) derived from the adsorption branch according to the NLDFT model for Ar adsorption at -186 °C in cylindrical pores of zeolite materials.

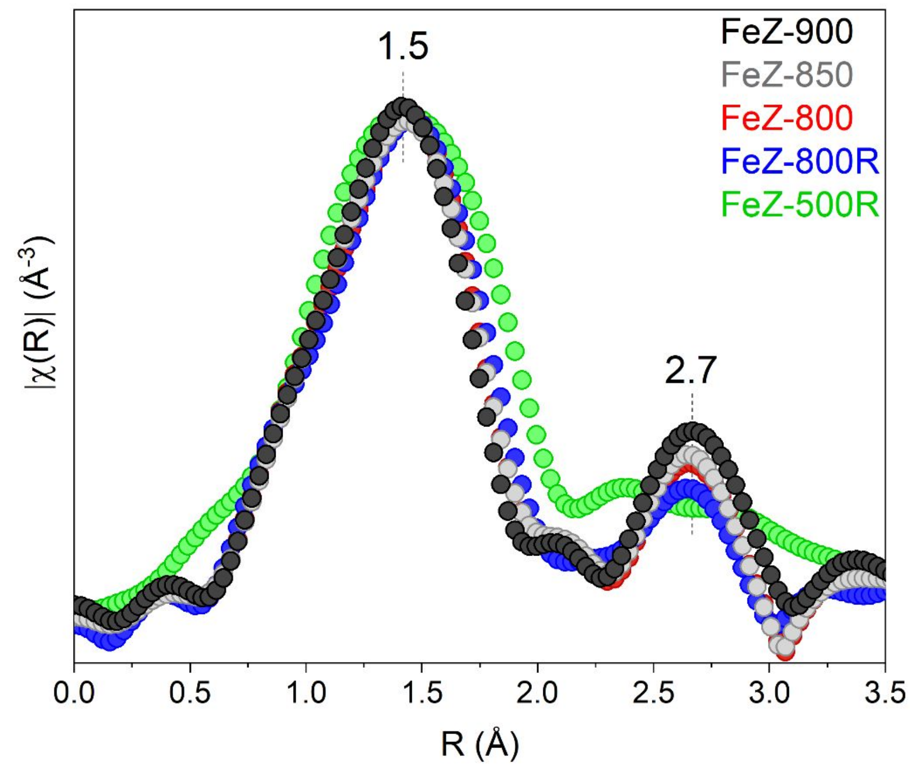

Figure S4. k^2-weighted EXAFS spectra of the Fe-ZSM-5 catalysts. The most intense scattering paths are indicated by vertical dashed lines.

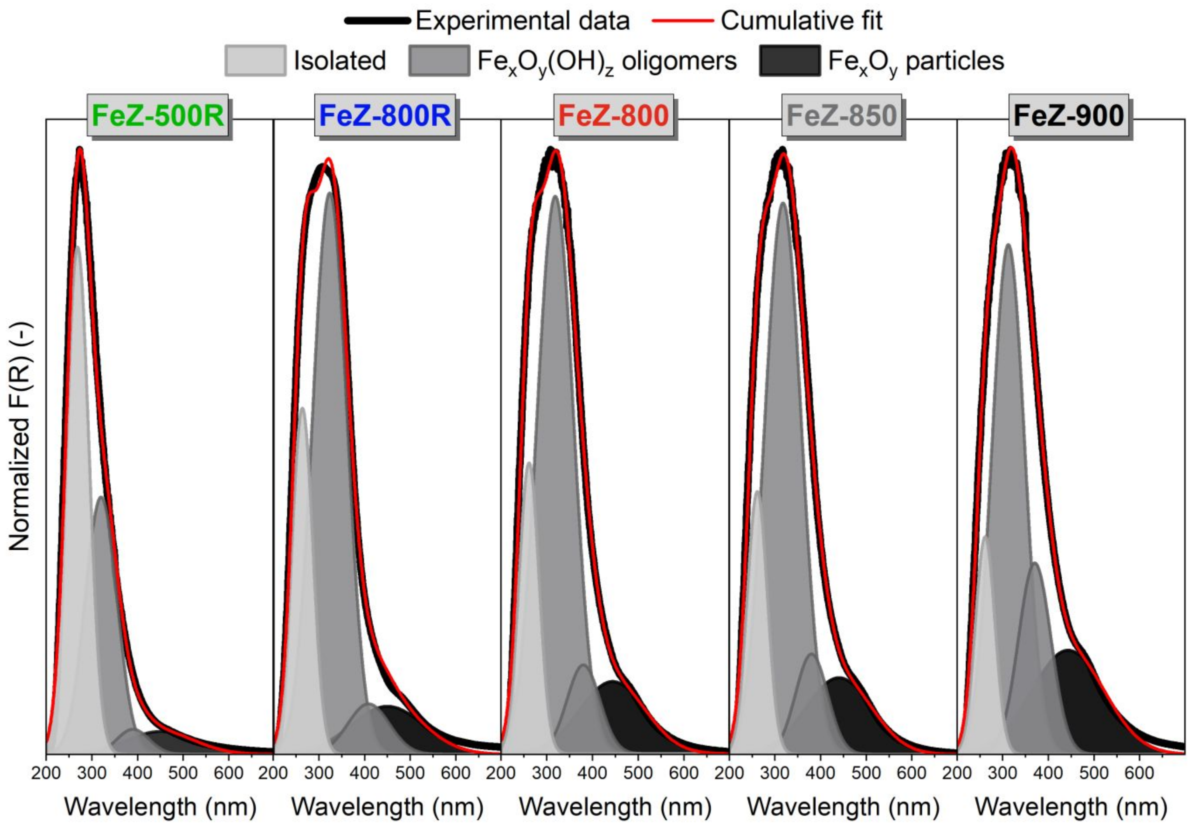

Figure S5. Normalized DRUV spectra of the dehydrated Fe-ZSM-5 catalysts. Individual Gaussian bands of the fitted spectra and cumulative fits are reported. The results of the fits are reported in Table S2.

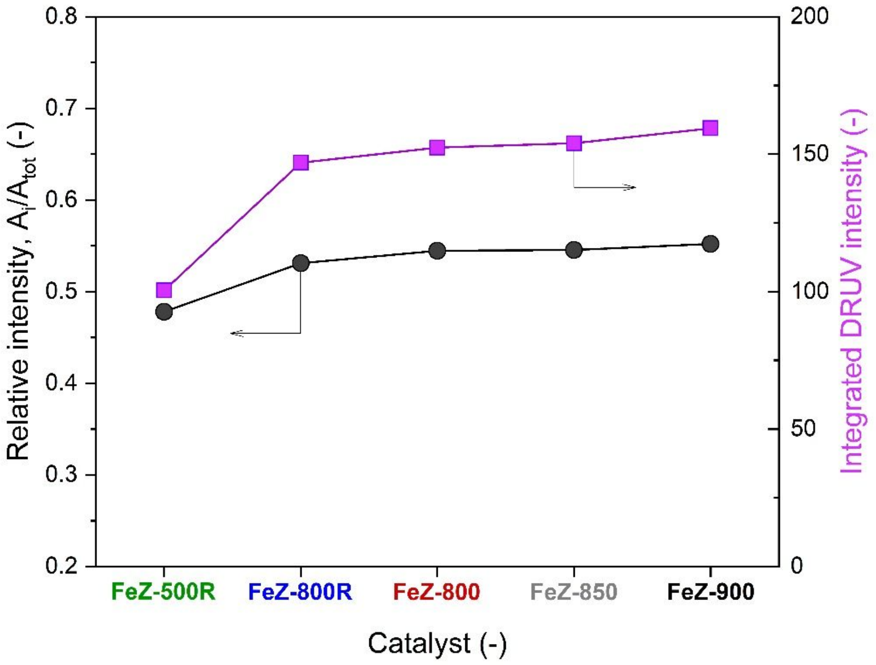

Figure S6. Contribution of oligomeric and hematite-like particles expressed as relative intensity (left-axis) to the total integrated intensity of the UV spectrum between 200 and 800 nm (right-axis) for the five Fe-ZSM-5 samples (see Figure S5 and Table S2).

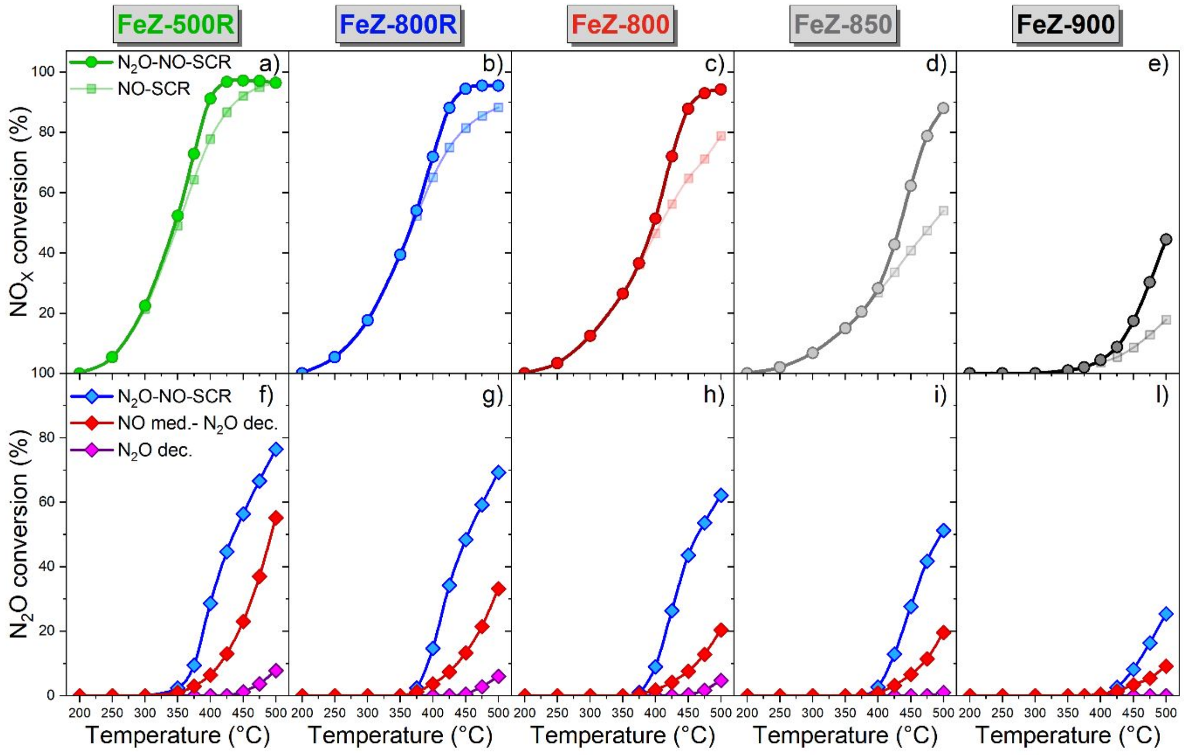

Figure S7. NO_x conversion under N_2O-NO-SCR and NO-SCR conditions (a-e) and N_2O conversion under N_2O-NO-SCR, NO-mediated N_2O decomposition and N_2O decomposition conditions (f-l). Feed compositions are reported in Table 1.

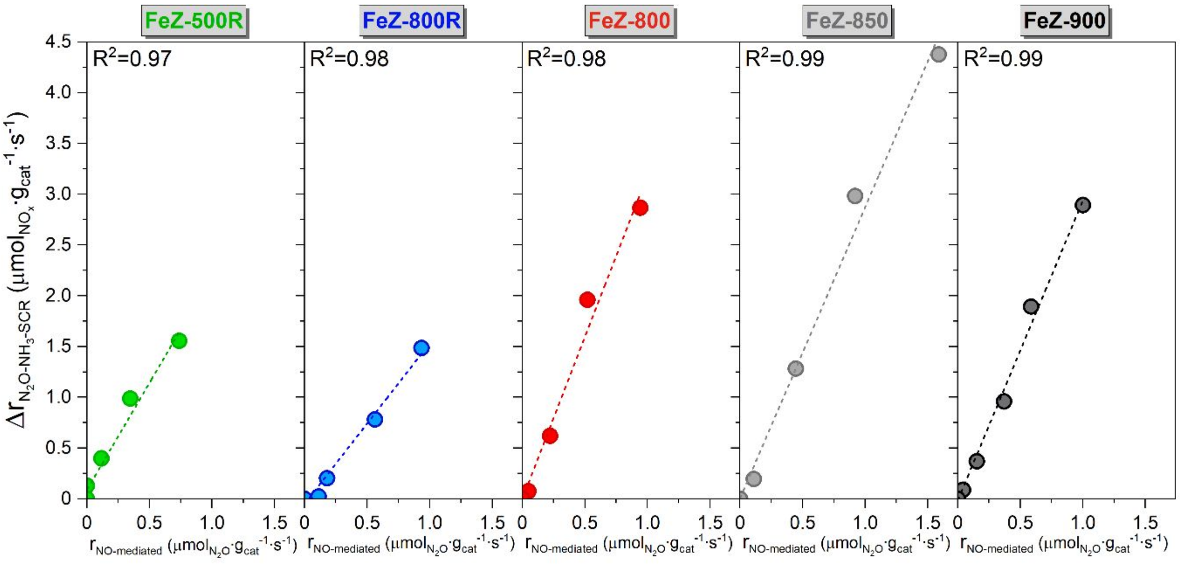

Figure S8. Relationship between the promotion of the NO-SCR reaction rate in the presence of N_2O ($\Delta r_{N_2O-NO-SCR}$) and the reaction rate for N_2O conversion under NO-mediated N_2O decomposition conditions ($r_{NO-mediated}$) at 400 °C. Experimental data (o) and linear fits (---) are reported. For each catalyst, the experimental points lying under limiting conditions have not been considered (see Figure 4b). These are: 500, 475, 450 and 425 °C (FeZ-500R), 500, 475 and 450 °C (FeZ-800R), 500 and 475 °C (FeZ-800), 500 °C (FeZ-850).

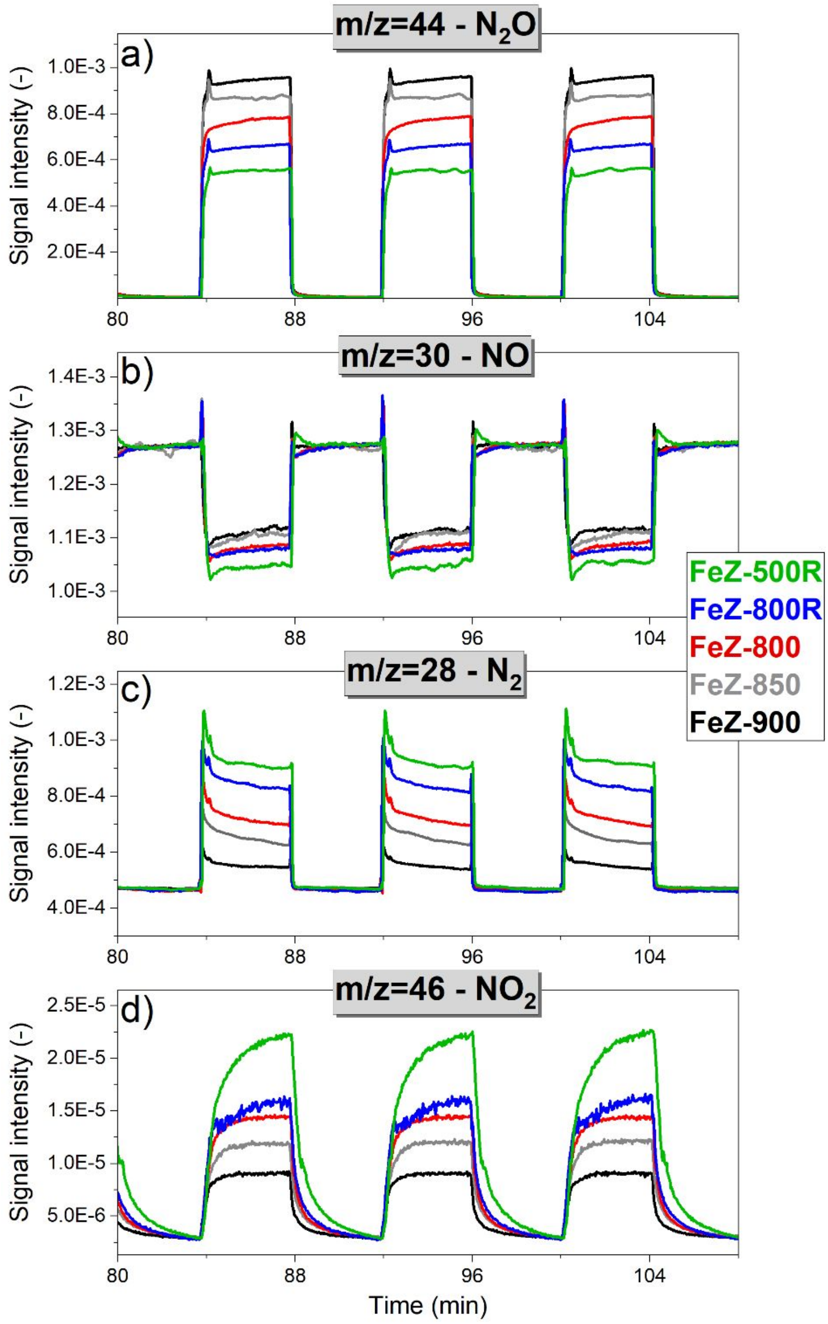

Figure S9. On-line MS signals related to N_2O (a), NO (b), N_2 (c) and NO_2 (d) during repeated pulses of 1000 ppm N_2O/Ar in a constant flow of 1000 ppm NO/Ar at 400°C while recording *operando* DRIFT spectra (see Figure 6).

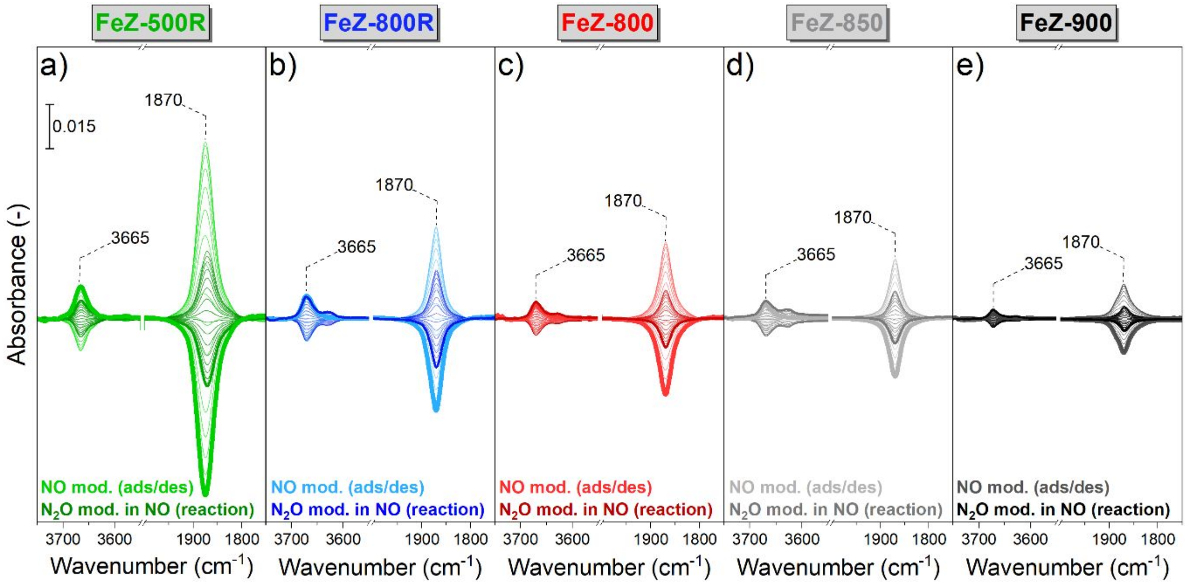

Figure S10. Phase-resolved DRIFT spectra during 4-min pulses of i) 1000 ppm N_2O/Ar in a constant flow of 1000 ppm NO/Ar or ii) 1000 ppm NO/Ar in Ar at 400 °C. The bold spectra (φ^{PSD} = 120°) are drawn to guide the eye.

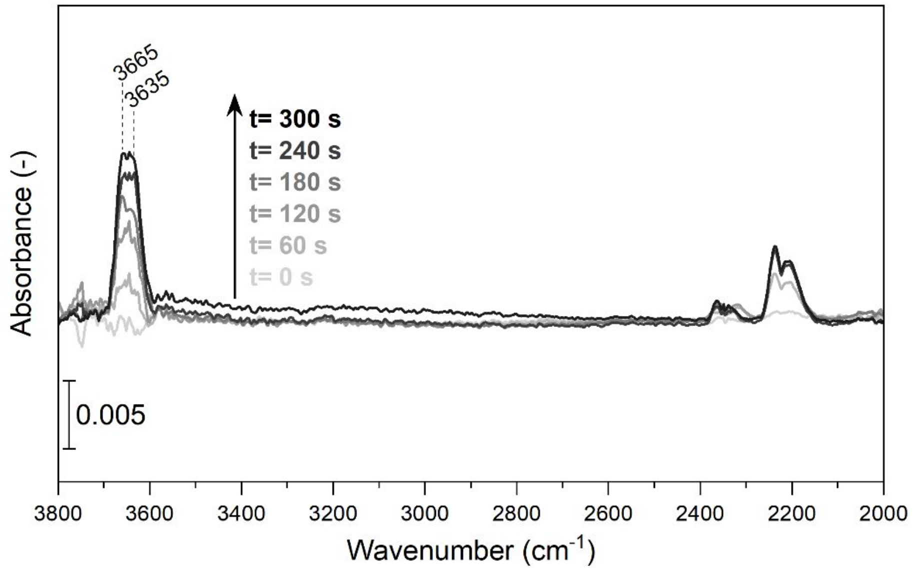

Figure S11. *In situ* DRIFT spectra of dehydrated FeZ-500R during addition of 1000 ppm N_2O/Ar in a constant flow of Ar at 400 °C.

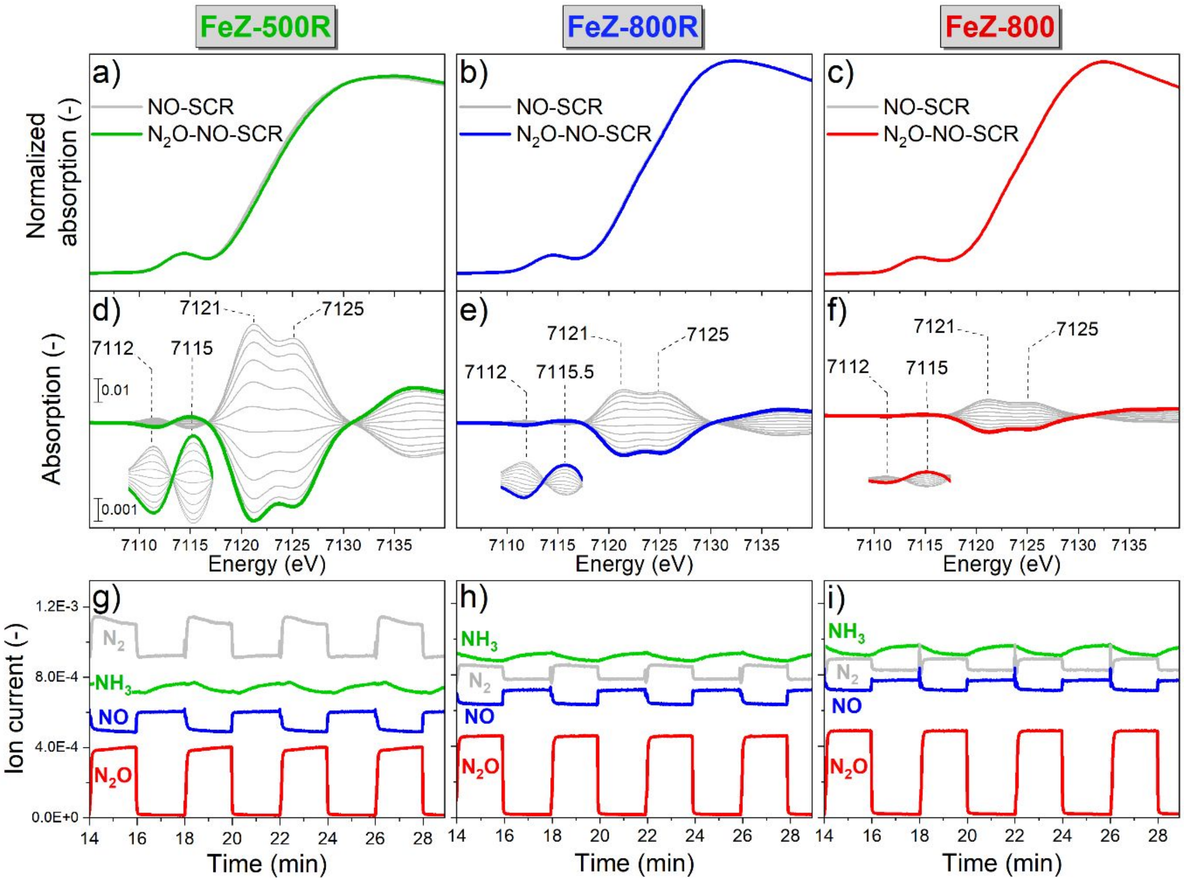

Figure S12. Averaged time-resolved (a, b, c) and phase-resolved (d, e, f) *operando* XAS spectra of FeZ-500R, FeZ-800R and FeZ-800 during 2-min pulses of 1000 ppm N_2O/Ar in a gas feed of 1000 ppm NO, 1000 ppm NH_3 and 3 vol% O_2/Ar at 400 °C and corresponding on-line MS signals (g, h, i). The bold spectra ($\varphi^{PSD} = 150°$) in d), e) and f) are drawn to guide the eye.

Supplementary References

(1) Buttignol, F.; Garbujo, A.; Biasi, P.; Rentsch, D.; Kröcher, O.; Ferri, D. Effect of an Al_2O_3-Based Binder on the Structure of Extruded Fe-ZSM-5. *Catal. Today* **2022**, *387*, 207-215
